# Construction of a Three-Color Prism-Based TIRF Microscope to Study the Interactions and Dynamics of Macromolecules

**DOI:** 10.3390/biology10070571

**Published:** 2021-06-23

**Authors:** Max S. Fairlamb, Amy M. Whitaker, Fletcher E. Bain, Maria Spies, Bret D. Freudenthal

**Affiliations:** 1 Department of Biochemistry and Molecular Biology and Department of Cancer Biology, University of Kansas Medical Center, Kansas City, KS 66160, USA; mfairlamb@kumc.edu (M.S.F.); awhitaker@kumc.edu (A.M.W.); 2 Department of Biochemistry and Molecular Biology, University of Iowa Carver College of Medicine, 51 Newton Road, Iowa City, IA 52242, USA; fletcher-bain@uiowa.edu (F.E.B.); maria-spies@uiowa.edu (M.S.)

**Keywords:** single-molecule TIRF, prism, single-molecule FRET, smFRET, construction guide, protein–DNA complexes, DNA repair complexes, transcription complexes

## Abstract

**Simple Summary:**

Prism-based single-molecule total internal reflection fluorescence (prismTIRF) microscopes are excellent tools for studying macromolecular dynamics and interactions. Here, we provide an easy-to-follow guide for the design, assembly, and operation of a three-color prismTIRF microscope using commercially available components with the hope of assisting those who aim to implement TIRF imaging techniques in their laboratory.

**Abstract:**

Single-molecule total internal reflection fluorescence (TIRF) microscopy allows for the real-time visualization of macromolecular dynamics and complex assembly. Prism-based TIRF microscopes (prismTIRF) are relatively simple to operate and can be easily modulated to fit the needs of a wide variety of experimental applications. While building a prismTIRF microscope without expert assistance can pose a significant challenge, the components needed to build a prismTIRF microscope are relatively affordable and, with some guidance, the assembly can be completed by a determined novice. Here, we provide an easy-to-follow guide for the design, assembly, and operation of a three-color prismTIRF microscope which can be utilized for the study of macromolecular complexes, including the multi-component protein–DNA complexes responsible for DNA repair, replication, and transcription. Our hope is that this article can assist laboratories that aspire to implement single-molecule TIRF techniques, and consequently expand the application of this technology.

## 1. Introduction

Prism-based single-molecule total internal reflection fluorescence (prismTIRF) microscopy is becoming ever-more popular due to its wide variety of applications. Multiple publications are available which outline the various capabilities of prismTIRF microscopes [1,2], the rationale behind fluorophore selection [3,4,5], the general assembly of a two-color prismTIRF microscope [6,7,8], and the basics of how to perform such experiments [3,9,10,11,12]. Although two-color TIRF microscopes are powerful tools, three-color TIRF microscopes allow more experimental flexibility by allowing users to perform three different combinations of dual fluorophore experiments. Additionally, the capability of three-color TIRF microscopes to simultaneously visualize three fluorophore markers are well suited to study systems involving more than two components. However, even with the currently available guides, building and operating a prismTIRF microscope without expert assistance can be a challenge. With this in mind, here we provide an easy-to-follow detailed guide for designing, assembling, and operating a three-color prismTIRF microscope which can be utilized for the study of biologically relevant macromolecular assemblies, including protein–DNA complexes.

This guide is broken into four main sections. The first section (Design) focuses on the rationale behind selecting certain key components of the prismTIRF microscope, as some components will vary depending on the experimental requirements. The second section (Construction) provides a detailed assembly procedure. This procedure is divided into three subsections: the excitation beam path (Section 3.1), which encompasses the components along the excitation path from the lasers to the microscope stage; the microscope stage (Section 3.2), which includes the inverted microscope and the components mounted to the stage; and the emission beam path (Section 3.3), which encompasses the components from the microscope to the camera. The third section (Operation) outlines the basics of prismTIRF microscope operation with a primary focus on the acquisition of total internal reflection, alignment of the excitation beams, and the assembly of bead slides and sample chambers. Finally, the fourth section (Application) provides pertinent examples of how TIRF microscopes have been utilized to study DNA replication, DNA repair, and DNA transcription.

## 2. Design

### 2.1. Prism-Based vs. Objective-Based TIRF

There are two main configurations of TIRF microscopes: objective-based and prism-based. In objective-based configurations, the excitation laser approaches the sample from below the microscope stage and passes through a specialized objective which directs the beam at the appropriate angle to induce TIRF [13,14]. As the excitation light approaches from underneath, objective-based configurations allow the top of the sample to be unobstructed and are ideal for imaging open samples such as culture dishes [7,13]. In prism-based configurations, the excitation laser is typically directed through a quartz prism positioned above the sample (see Section 3.2.2). Thus, in prismTIRF the surface of the quartz slide within the sample chamber is imaged, whereas the surface of the glass coverslip is imaged in objective-based configurations. This means that prismTIRF configurations can be used to image samples during active flow, which is challenging to perform using an objective-based microscope due to the pressure-induced warping of the coverslip. Additionally, prismTIRF allows for easier manipulation of the excitation beam trajectory and a slightly higher signal-to-noise ratio. Therefore, prismTIRF is well-suited for studying reactions that can be contained within a sample chamber, including interactions between fluorescently labeled macromolecules and surface-tethered substrates. Furthermore, prismTIRF microscopes are relatively simple to assemble and operate, which make them ideal for first-time users. Here, we focus on the assembly of a prismTIRF microscope. Resources regarding the construction of an objective-based TIRF configuration [1,7,15,16], including the cost-effective smfBox [8] which is capable of single-molecule Förster resonance energy transfer (smFRET) experimentation, are available at the associated references. 

### 2.2. Fluorescent Labels and Excitation Lasers

**Figure 1:**
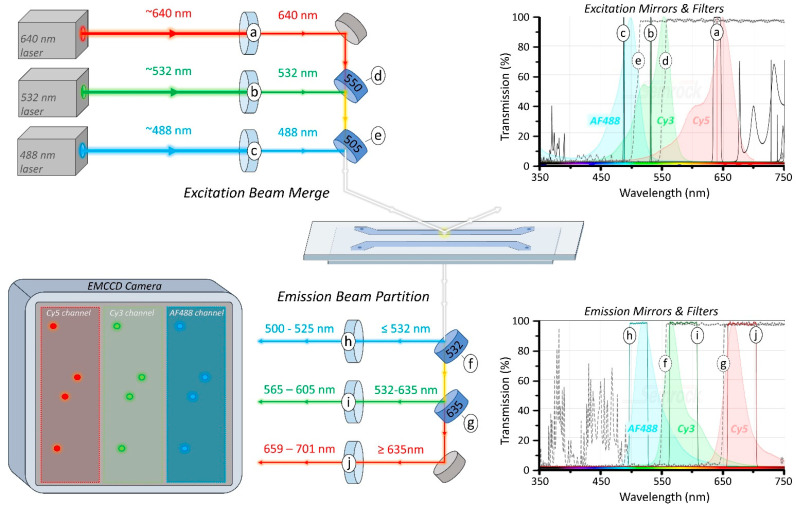
Dichroic mirror and filter scheme. Clean-up filters (a–c) block unwanted excitation energy from reaching the sample. The 640 nm beam passes through the back of the 550 nm longpass dichroic mirror (d) while the 532 nm beam is reflected from the front, merging the path of the two beams. To combine the 488 nm beam into the laser path, the 640/532 nm beam is passed through the back of the 505 nm longpass dichroic mirror (e) while the 488 nm beam is reflected from the front. Emission from AF488 is partitioned with a 532 nm longpass dichroic mirror (f), while the Cy3 and Cy5 emission are seperated with a 635 nm longpass dichroic mirror (g). Emission filters (h–j) are then used to eliminate bandwidths of light not pertaining to the intended fluorophore. The resulting emission channels are projected onto the electron multiplying charge-coupled device (EMCCD) camera such that they fill the detector space while not overlapping.

One of the first steps in designing a prismTIRF microscope is to decide on the fluorophores that will be utilized during experimentation. This will dictate the choice of lasers, mirrors and filters within the excitation path, and the mirrors and filters within the emission path. For experiments where different colored fluorophores will be simultaneously excited and imaged, it is ideal to use a series of fluorophores which have spectrally distinct excitation and emission spectra. This ensures that each excitation laser only excites the intended fluorophores and that the emission from each type of fluorophore can be easily distinguished (Figure 1). The SearchLight Spectra Viewer program provided by Semrock is a good resource for deciphering the overlap of excitation and emission spectra (https://searchlight.semrock.com, accessed on 5 March 2021) [17]. The brightness, stability, and Förster resonance energy transfer (FRET) characteristics of fluorophores are also important attributes to consider, and excellent resources are available which provide extensive details in this regard [1,4,18].

Three fluorophores were selected to be utilized in the prismTIRF microscope described here: Cyanine-5 (Cy5), Cyanine-3 (Cy3), and Alexa Fluor 488 (AF488). These are some of the more commonly used fluorophores for single-molecule TIRF experiments due to their stability, brightness, FRET capacity, and well-separated excitation/emission spectra (Figure 1). However, the infrared fluorophore Cyanine-7 [5], the environmentally sensitive MB543 [19], and the many Alexa Fluor variants have also been used with great success. Although this guide is written specifically with these fluorophores in mind, the rationale behind component selection described below applies to any set of chosen fluorophores.

The ideal excitation laser for a particular fluorophore has a wavelength which maximizes the excitation of the intended fluorophore while minimizing the excitation of others. The excitation spectra of AF488, Cy3, and Cy5 and the lasers chosen to excite them are shown in Figure 1. Although the excitation maxima of Cy3 is 554 nm, the optimal excitation wavelength in this case is 532 nm, since it excites AF488 and Cy5 less efficiently (Figure 1b). Similarly, 488 nm is the optimal excitation wavelength for AF488 excitation because it minimizes the excitation of Cy3 (Figure 1c). Although Cy3 is excited by 488 nm light to some degree, the Cy3 emission will be relatively dim compared to AF488 and can be accounted for during data analysis [16]. Typically, lasers rated with a maximum power-level of at least 50-100 mW are sufficient for TIRF imaging [9].

### 2.3. Longpass Dichroic Mirrors

To achieve an overlapping excitation field of all three lasers at the region being imaged, the paths of the three beams are merged before reaching the sample (Figure 1d,e). Additionally, since the camera cannot differentiate between wavelengths of light, the emission from each of the three fluorophores must be partitioned and projected onto separate regions of the detector surface (Figure 1f,g). Both the merging of the excitation beams and the partitioning of emission light are achieved using longpass dichroic mirrors, which reflect light below a cut-off wavelength while being transmissive to wavelengths of light above the cut-off [20]. In this section, we discuss the rationale behind selecting dichroic cut-offs and the mirror quality requirements at each position.

Merging the three excitation beams is performed using two longpass dichroic mirrors (Figure 1d,e). The first dichroic mirror is used to merge the two longest excitation beams and is positioned such that the longer wavelength beam (640 nm) passes through the back of the mirror, while the shorter wavelength beam (532 nm) reflects from the front. In order for the mirror to be transmissive for the longer wavelength and reflective of the shorter wavelength beam, a cut-off between the two wavelengths is selected (550 nm) (Figure 1d). The second dichroic mirror serves to merge the shortest wavelength beam (488 nm) with the other two and requires a cut-off wavelength between the shortest and second shortest beam (488/532 nm; 505 nm cut-off) (Figure 1e). Again, the two longer wavelength beams should pass through the back of the mirror and align with the path of the reflected beam, merging the three excitation beams.

Emissions from the three fluorophores are partitioned using two dichroic mirrors positioned within an Optosplit III filter cube (see Section 3.3) (Figure 1f,g). The ideal cut-off for these dichroic mirrors will reflect as much emission from the intended fluorophore as possible while being transmissive to the emission of the other fluorophore(s). For example, AF488 emission can range from ~490 nm to ~625 nm and partially overlaps with the emission spectra of Cy3 (Figure 1f). To reflect the most amount of AF488 emission while minimizing the reflection of Cy3 and Cy5 emission, a dichroic mirror with a cut-off of 532 nm is used. The second dichroic mirror within the Optosplit III is used to reflect emission of the fluorophore with the second shortest emission wavelength (Cy3) (Figure 1g). In this case, a cut-off of 635 nm is selected, which will reflect nearly all Cy3 emission (532–635 nm) while being transmissive to the majority of Cy5 emission. After the emissions are separated, bleed over emission from fluorophores with overlapping emission spectra are attenuated using emission bandpass filters (see Section 2.4) (Figure 1h–j).

Finally, using mirrors with the appropriate flatness is critical to acquiring clear images. When images are reflected off of an insufficiently flat mirror, the location of the focal plane can shift along the optical axis and the focus spot can be distorted, causing the image to become blurry [21]. The emission light that is projected onto the camera is a two-dimensional image and contains pertinent X–Y information. Thus, mirrors within the emission path must be of high enough quality to minimize image distortion. The Optosplit III supports 26 mm × 38 mm rectangular mirrors up to 3 mm in thickness. The manufacturer recommends using 2 mm thick mirrors with a flatness of at least λ/2. However, we have found that 1 mm thick BrightLine^®^ dichroic mirrors with a reflected wavefront error of <1λ P-V @ 632.8 nm have been sufficient to yield a clear image in our setup. That said, we suggest contacting the supplier directly to discuss their recommendations before purchasing. Conversely, excitation lasers do not contain pertinent X–Y information, so the quality of mirrors within the excitation path is of less consequence, as long as the wavefront error is <6λ P-V @ 632.8 nm [21].

### 2.4. Bandpass Filters

Bandpass filters are only transmissive for certain bandwidths of light and are used in both the excitation and emission paths to eliminate undesired wavelengths of light. In the excitation path, laser clean-up filters with narrow transmissive bands are used to exclude any erroneous excitation energy from reaching the sample (Figure 1a–c). This helps prevent indiscriminate fluorophore excitation and minimizes background noise. Additionally, after the emission from each fluorophore is partitioned, selective bandpass filters are used to block excitation light and bleed over emission from other fluorophores from reaching the camera (Figure 1h–j). In some cases, it is possible to select emission filters which exclude nearly all emissions from unintended fluorophores, such as the emission filters used here for AF488 and Cy5 (Figure 1h,j). However, for fluorophores with an emission spectrum which is overlapped by another fluorophore, such as Cy3, a filter should be selected which has a transmissive bandwidth that maximizes the percentage of intended light over erroneous light (Figure 1i). This can be estimated using the Optimization Calculator within the SearchLight Spectra Viewer provided by Semrock [17]. Additionally, we suggest contacting the supplier for recommendations to select the optimal arrangement of mirrors and filters. After selecting the optimal dichroic mirrors and bandpass filters to acquire the best possible emission separation from the three fluorophores, the remaining bleed through can be corrected for during analysis [5,16,22].

## 3. Construction

### 3.1. Excitation Beam Path

#### 3.1.1. Safety Considerations

The lasers used in this setup are rated Class 3B, which can heat skin and materials but are not considered a burn hazard. However, at 5 to 50 milliwatts of power, a Class 3B laser does pose a moderate risk of eye injury, especially if the laser light remains on one spot on the retina long enough for heat to build up to injurious levels. Thus, it is important to take measures to prevent direct eye exposure, especially during construction when there is a high risk of inadvertent eye exposure. Laser safety goggles specific for the wavelengths of light being used should be worn during construction while the excitation lasers are active (https://lasersafetyindustries.com, accessed on 5 March 2021). In order to see the laser beam while working, the goggles should have an OD of 2–3, which is high enough to protect the eyes but will not block 100% of the laser’s light. Additionally, black out curtains or a foam board perimeter should be installed to contain reflected laser light within the setup during everyday operation. Finally, in most cases, the Class 3B lasers must be registered with the institutional laser safety and the appropriate safety warning signage will need to be posted.

#### 3.1.2. Optical Table and Operating Space

**Figure 2:**
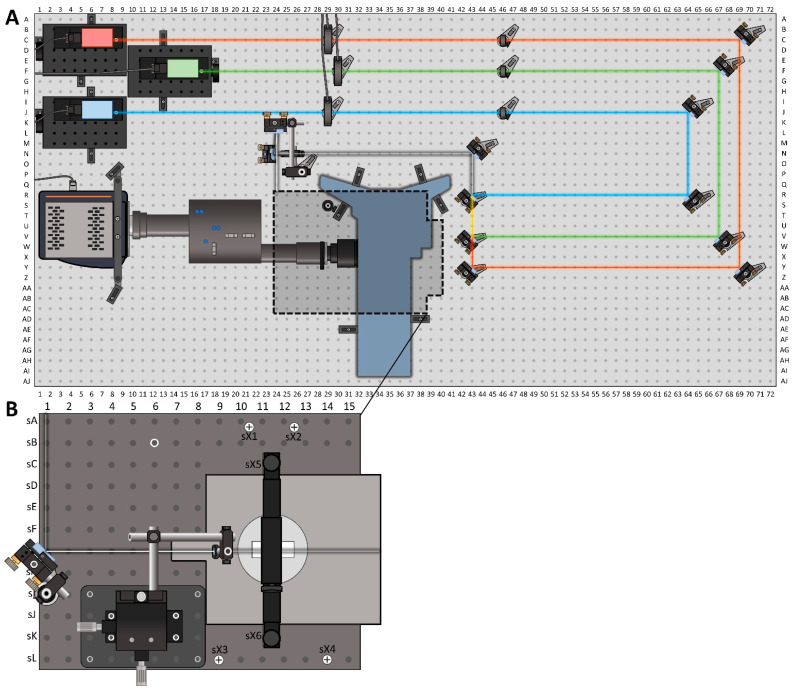
Layout of the prismTIRF microscope. (**A**) The excitation laser beams (A1–M18) pass through shutters (A28–K31) and clean-up filters (B45–K47) before being leveled with a two-mirror turn (A64–AA72). The beam paths are then merged using two dichroic mirrors (Q41–AA44). The combined beams are redirected (N43) to a periscope (J22–P28) that supports two mirrors which elevate the beam to the level of the stage. (**B**) The microscope stage is fitted with a custom aluminum breadboard (sA1–sL15) that is attached via the front (sX3 and sX4) and rear (sX1 and sX2) stage adapter pieces, and stabilized by a support post that spans between sB6 of the stage breadboard and S29 of the optical table. The beam is reflected from the top mirror of the periscope to the stage mirror (sG1), travels squarely over the sG2–sG6 holes, and passes through a plano-convex lens (sG9) attached to an XYZ linear stage (sI3–sL8). The beam then passes through a quartz prism (sG11–sG12) positioned on top of the sample chamber. The prism is secured in place with an overhead arm mounted to the stage breadboard (sX5–sX6). The emission path passes through the inverted microscope (P28–AJ40) and enters the Optosplit III (R10–Y29), which is attached to the left port via the C-mount camera adapter (V29–Y32). Dichroic mirrors and emission filters within the Optosplit III (V18–X22) partition the emission from each fluorophore before the image is projected onto the camera (O1–AB10).

The optical Table (Table 1, #1,2) is the foundation of the prismTIRF microscope (supplemental Figure S1A). To provide ample room for the mounting of all components, we recommended using a table that is 3’ by 6’ and fitted with ¼”-20 NC tap holes on a 1” grid (Figure 2A). However, the layout depicted in Figure 2 could be accommodated to fit on a smaller table by reducing the distance between components in the excitation beam path. The main constraint regarding space is the fixed components within the emission path (i.e., inverted microscope, Optosplit III, and camera). Thus, if space is limited, we suggest using a low-profile alternative to the Optosplit III. The table should be constructed in a low-traffic isolated area to minimize vibrations and potential table bumps which can disrupt image quality and mirror alignment. Additionally, the location must be capable of being completely darkened during microscope operation and, if needed, should be surrounded by black out curtains to reduce aberrant light noise and minimize unintended laser exposure to non-users. Over time, dust particles can settle on the optical components, which will require regular cleaning. Thus, it can be beneficial to minimize the amount of dust and particles in the air by running an air filtration device (Table 1, #82) nearby and/or installing air filters on nearby vents. Finally, it is recommended to assemble an overhead table shelf (Table 1, #3) over the optical table to allow the off-table storage of power supplies and provide easy access to the laser and shutter controllers.

#### 3.1.3. Excitation Lasers, Shutters and Clean-Up Filters

The three-color prismTIRF microscope described here is fitted with 488 nm, 532 nm, and 640 nm excitation lasers (Table 1, #16–18) allowing for the simultaneous excitation of AF488, Cy3, and Cy5 fluorophores (supplemental Figure S1B). To ensure thermal stability of the lasers, each can optionally be mounted to a heat sink (Table 1, #19) which will minimize temperature fluctuations during operation. The three laser/heat sink assemblies are attached to vertical translation stages (Table 1, #20) and secured in place using L-shaped clamps (Table 1, #11). This allows for coarse adjustment to the height of the beam off the table surface. As shown in Figure 2A, each laser assembly is positioned such that the 640 nm, 532 nm, and 488 nm beams travel, respectively, over the “C”, “F”, and “J” rows of tapped holes in the table. Using the beam height measurement tool (Table 1, #72), the height of each vertical translation stage is adjusted such that the 640 nm, 532 nm, and 488 nm beams are 8” off the table surface when they reach the C69, F67, and J64 holes, respectively (Figure 2A). Finally, each excitation laser is wired to an adjustable power source (Table 1, #21) positioned on the overhead shelf (supplemental Figure S1C). Optionally, the adjustable power source can be connected to a nearby computer and controlled using Coherent Connection software provided by the manufacturer.

The excitation lasers require time to warm up and shut down (~5 min), so shutters are incorporated in the beginning of each excitation laser’s path to allow for quick and independent toggling of the excitation beams (supplemental Figure S1B). Each shutter is attached to a 4” post and an adjustable post holder and secured to the table using a pedestal base adaptor and slotted clamping fork such that the beam passes through the center of the aperture (Table 1, #22, #6, #8–10) (Figure 2A, C29, F30, J29). Optionally, these shutters can be outfitted with “Electronic Sync” that enables computer-controlled shutter operation allowing for Alternating Laser Excitation (ALEX) experiments [23,24]. Lastly, each shutter is hardwired to a shutter driver switch box (Table 1, #23) positioned on the overhead table shelf above the inverted microscope (supplemental Figure S1C).

At times, excitation lasers can emit erroneous energy and unintended wavelengths of light. Clean-up filters minimize the amount of this undesired excitation energy from reaching the sample (Figure 1a–c). Each wavelength-specific clean-up filter (Table 1, #24–26) is secured inside a fixed lens mount (Table 1, #14) using a 1/2” spanner wrench (Table 1, #68) and attached to a 6” post, adjustable post holder, and pedestal base adapter (Table 1, #7–10). The clean-up filter assemblies are then mounted to the optical table immediately following the shutters using slotted clamping forks such that the beams pass through the center of the filters (Figure 2A, C46, F46, J46) (supplemental Figure S1D).

#### 3.1.4. Leveling and Merging the Laser Beam Paths

After the beams pass through the shutters and clean-up filters, they each are reflected from two broadband mirrors that together turn the beams 180°. This two-mirror turn is a tactic that allows for the trajectory of each beam to be fine-tuned, such that they travel level with the surface of the table. To set up the two-mirror turn, six mirror assemblies (two for each beam) containing broadband mirrors (Table 1, #27) are constructed using clear-edge mirror mounts, 6” posts, adjustable post holders, and pedestal adaptors (Table 1, #7–10 and #13). The mirror assemblies are then mounted such that the beams strike the center of the mirrors directly above the tapped hole, as indicated in Figure 2A (C69, Y69, F67, V67, J64, and R64), 8” above the table surface and are reflected at a 90° angle.

Leveling each beam requires the use of two irises positioned after the second mirror (supplemental Figure S2). Each iris is attached to a 6” post, adjustable post holder, and a ¼”-20 machine screw (Table 1, #7,8 and #69,70), and adjusted such that the very center of aperture is 8” off the surface of the table using a ruler or a beam height measuring tool (Table 1, #72) (supplemental Figure S2A). A slip-on post collar (Table 1, #71) can then be attached to the iris assembly, which will allow the post to be rotated within the adjustable post adapter without altering the height of the iris. Starting with the shortest wavelength beam path, the two 8” irises are then mounted directly into holes of the table, via the ¼”-20 screw, just beyond the second mirror (R62 and R46 for 488 nm beam) (supplemental Figure S2B,C). Optionally, if the microscope has not already been placed on the table, the second iris can be placed at the edge of the table (R1) to maximize the distance between the two irises, which will increase the precision of the laser leveling. 

With the two irises in place, the beam can be “walked” into position by adjusting the first and second mirrors iteratively. To start, the apertures of both irises are narrowed and the first mirror is adjusted so that the beam reflects off the second mirror and passes through the very center of the nearest iris (supplemental Figure S2B). Then, the aperture of the nearest iris is opened slightly, and the second mirror is adjusted so the beam passes through the center of the far iris (supplemental Figure S2C). This process is repeated, alternating between adjusting the first mirror so the beam passes through the center of the narrowed near iris, and adjusting the second mirror so the beam passes through the center of the narrowed far iris, until the beam simultaneously passes cleanly through the very center of both narrowed irises. With the leveling complete for the 488 nm beam, the leveling process is repeated for the 532 nm and then 640 nm beams, moving the irises to V65 and V46 for the 532 nm beam and Y67 and Y46 for the 640 nm beam. After all three beams are leveled, each beam will be on a level plane 8” off the table surface. For additional help, video tutorials describing the process of walking a beam into alignment can be found on the Thorlabs website (https://www.thorlabs.com/, accessed on 5 March 2021). 

The beam paths are combined using a broadband mirror and two longpass dichroic mirrors (Figure 1d,e). To set up the beam merging mirrors, the two 8” tall irises are first mounted directly into the P43 and A43 holes and both apertures are narrowed (Figure 2A). A mirror assembly (Table 1, #7–10 and #13) containing the 505 nm cut-off dichroic mirror (Table 1, #28) is then placed on the table such that the 488 nm beam reflects at a 90° angle off the front face of the mirror over the R43 hole and passes through the very center of the two narrowed irises. After the 505 nm mirror is in position, a mirror assembly (Table 1, #7–10 and #13) containing the 550 nm cut-off dichroic mirror (Table 1, #29) is then placed on the table such that the 532 nm beam reflects at a 90° angle off the front face of the mirror over the V43 hole, passes through the back of the 505 nm dichroic mirror, and passes through the very center of the two irises. Finally, a mirror assembly (Table 1, #7–10 and #13) containing a broadband mirror (Table 1, #27) is placed on the table such that the 640 nm beam strikes the center of the mirror over the Y43 hole, is reflected at a 90° angle, and passes through the back of the 550 nm mirror, 505 nm mirror, and then through the very center of both irises. The additional space between the 505 nm and 550 nm dichroic mirrors provides more room for the user to access the adjustment knobs on the 505 nm mirror mount (see Section 4.2). When all three beams pass through the center of both irises, the beams have been successfully merged. Note that following the order described above for the incorporation of the mirrors will make this step significantly easier, as chromatic aberrations in the dichroic mirrors could alter the trajectory of the transmitted beam(s).

#### 3.1.5. Beam to Stage Path

After the excitation beams are merged, they are directed to the stage of the inverted microscope using a series of broadband mirrors (supplemental Figure S3A). Note that from this point forward, only one of the three beams should be on while setting up the remainder of the excitation beam path. We recommend using the beam which will be used most often during operation to establish this path and/or the mid-wavelength beam. This is because the other beams will require periodical realignment to match the beam used to establish the excitation path (see Section 4.2), but the path of the established beam will remain unchanged after construction. Here, the mid-wavelength beam (532 nm) was used to set up the remainder of the excitation path.

A broadband mirror assembly (Table 1, #7–10, #13, #27) at N43 is used to direct the beam behind the microscope and it is positioned such that the beam is reflected through the center of two 8” tall irises mounted into the N41 and N14 holes (Figure 2A). The beam is then elevated to the height of the microscope stage using two broadband mirrors attached to an 18” periscope (supplemental Figure S3B,C). The periscope is assembled from three 6” optical posts and secured to the table near the O26 hole using an adjustable post holder, pedestal base adaptor, and a slotted clamping fork (Table 1, #7–10). The two broadband mirrors are mounted within clear-edge mounts, attached to 2” posts, and then each is secured to the 18” post using two right angle clamps and a 4” post (Table 1, #4, #6, #12,13, #27). The bottom mirror is positioned such that the beam is directed 90° vertically, parallel with the 18” post, and the top mirror is positioned to redirect the beam 90° horizontally toward the front edge of the table. The exact positioning of the top mirror will be determined in Section 3.2.2, after the inverted microscope is assembled. An alternative option to the periscope is to use 30 mm cage optics. Although the cage optics will incur an additional cost, it will save time in aligning the excitation beam to the stage.

### 3.2. Microscope Stage

#### 3.2.1. Inverted Microscope and Stage Breadboard

The inverted microscope is placed on the table with the front bottom edge of the microscope sitting roughly 2” from edge of the table and centered between the 34th and 35th column of tapped holes (Figure 2A, supplemental Figure S1). Securing the microscope in place are six L-shaped clamps at holes AE31, T30, P32, P38, S40, and AD38. Once the inverted microscope is secured in place, the microscope components (Table 1, #30–39) can be assembled according to the IX73 user manual.

A series of components mounted to the microscope stage are used to direct the excitation beam into the sample chamber. To secure these components to the stage, a custom-made stage breadboard (Table 1, #42) with ¼”-20 NC tapped holes in a 1” grid is attached to the top of the inverted microscope. Before the stage breadboard can be attached, two custom-made stage adapters (Table 1, #40,41) are secured to top of the inverted microscope via machine screws (Table 1, #52, #54) (supplemental Figure S4). The stage breadboard can then be bolted to the microscope via the stage adapters at position sX1–sX4 (Table 1, #51, #53) (Figure 2B, supplemental Figure S4B,C). These custom components can be commissioned from a local machine shop, using the design files provided on our lab website (https://www.freudenthallab.com/, accessed on 5 March 2021). Finally, to provide additional stability to the stage breadboard, a support beam built from a 4” and 6” post, adjustable post holder, pedestal base adaptor, and a slotted clamping fork (Table 1, #6–10) is mounted between the optical table (S29) and the breadboard stage (sB6) (supplemental Figure S4C). 

#### 3.2.2. TIR Angle and the Stage Mirror

**Figure 3:**
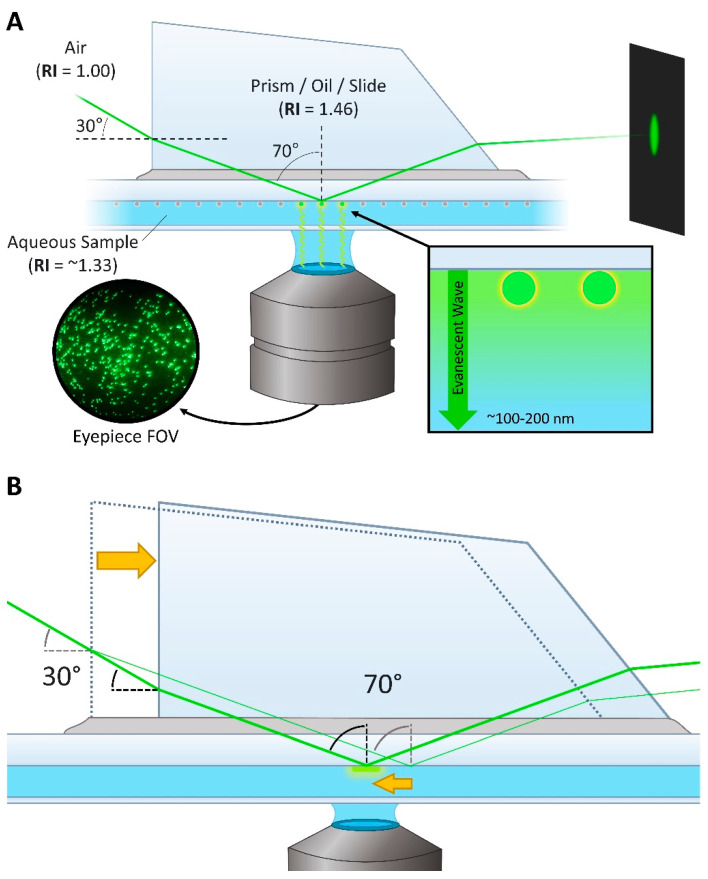
Trajectory of excitation beam through the prism. (**A**) The excitation beam enters the front face of the quartz prism at an incident angle of ~30°. The difference in refractive index between the air and quartz results in a 20° angle of refraction. The quartz prism, oil, and quartz slide have similar refractive indices, which minimizes further refraction of the beam. The beam approaches the interface of the quartz slide and aqueous sample at a ~70° incident angle, resulting in total internal reflection of the beam and the generation of an evanescent wave which emanates 100–200 nm into the sample. When TIRF is achieved at the surface of the quartz slide, the reflected beam should produce a symmetric circle on the far wall. (**B**) The prism is used to adjust the trajectory of the excitation beam. Sliding the prism horizontally alters the height at which the beam enters the prism, which shifts where the beam strikes within the sample chamber while maintaining the proper 70° incident angle.

In prismTIRF microscopy, the excitation beam is directed into the sample chamber at a particular incident angle which results in the beam being totally reflected from the slide/aqueous sample interface (Figure 3A) [13,25,26]. This total internal reflection (TIR) generates an evanescent wave that penetrates ~100–200 nm into the sample chamber, only exciting the fluorophores located within this thin band [14]. The penetration depth of the evanescent wave depends on the incident angle of the beam, the refractive index (RI) of the two mediums at the interface (i.e., the quartz slide and aqueous sample), and the wavelength of the beam [27,28]. Evanescent waves produced from longer wavelength beams and smaller incident angles will penetrate deeper into the sample. For a quick tutorial on TIR angles and evanescent wave penetration depths, we recommend referencing the evanescent wave penetration depth java tutorial provided by Olympus [29]. In most cases, simply knowing that the system is in the TIRF imaging mode and having a rough estimate of the penetration depth is adequate. However, if quantitative measurements of the penetration depth are desired, there are ways to determine these values [27,28].

To induce TIR at the interface of the quartz slide (RI = 1.46) and aqueous sample within the chamber (RI = ~1.33), the incident angle of the co-aligned 488 nm, 532 nm, and 640 nm beams should be between 68–71° (Figure 3A) [6,14,27,28]. At 70°, the 488 nm, 532 nm, and 640 nm beams will generate evanescent waves which penetrate roughly 105 nm, 114 nm, and 138 nm into the sample, respectively [14]. Due to the difference in RI between the air (RI = 1.0) and quartz prism (RI = 1.46), the beam will refract upon penetrating the quartz prism before reaching the slide/sample interface. Thus, the beam should enter the quartz prism at a 28–33° incident angle to induce the 68–71° incident angle at the slide/sample interface.

A broadband mirror positioned on the stage breadboard (named the stage mirror) is used to direct the co-aligned excitation beam into the sample chamber (supplemental Figure S5). The height at which the beam should strike the stage mirror to induce the 30° incident angle at the prism face is dependent on the horizontal distance between where the beam strikes the mirror and the prism face. In this particular setup, the beam should traverse directly over the sA1–sF1 holes of the stage breadboard and reflect off the stage mirror directly above sG1, which is approximately 26.5 cm (10.4”) from where the prism will be positioned. Considering the surface of the stage breadboard is ~2 cm (0.8”) above the microscope stage, the beam should strike the stage mirror 13.5 cm (5.3”) above the surface of the stage breadboard, totaling 15.5 cm (6.1”) of vertical difference to induce a 30° incident angle at the prism face.

To set up the stage mirror, two irises with centers at 13.5 cm (5.3”) high are placed in the sA1 and sL1 holes of the stage breadboard, and the top mirror on the periscope is adjusted such that the beam travels through the center of both irises (supplemental Figure S5A). This will require multiple iterative adjustments to both the bottom and top mirror of the periscope to achieve the proper beam trajectory. When finished, the irises are removed and a broadband mirror is inserted into a clear-edge mirror mount, attached to a 2” post, combined to a 6” post using a right-angle clamp, and then secured to the stage breadboard using a 1” post holder and a slotted clamping fork (Table 1, #4, #7, #10, #12,13, #15, #27) (supplemental Figure S5B). The stage mirror is then positioned over the sG1 hole in the stage breadboard such that the beam is reflected squarely over the sG2–sG6 holes and strikes the microscope stage between the overhead arm mounting holes sX5 and sX6 (Figure 2B, supplemental Figure S5C,D). To observe the position where the beam strikes the stage, a glass microscope slide covered with opaque tape can mounted to the stage. To ensure that the beam is traveling squarely over the sG2–sG6, two 6” posts can be inserted into the sG2 and sG6 holes of the stage breadboard. If the beam is square over the holes, it should strike the center of the sG2 post, and after the sG2 post is removed, the beam should also strike the center of the sG6 post. Alternatively, irises can be used instead of 6” posts for a more precise calibration of the beam trajectory.

#### 3.2.3. Focusing Lens and Prism Assemblies

Optimal TIRF imaging typically requires making small adjustments to the position and diameter of the illuminated field during operation. These adjustments are made using a plano-convex lens (Table 1, #55) and a quartz prism (Table 1, #73), which can be used to focus the beam and fine tune the beam trajectory. Before setting up the prism and lens, the objective will need to be adjusted to the proper height. This can be performed by placing a bead slide (see Section 4.1 below) on the stage and focusing on the inside edge of the adhesive within the chamber (supplemental Figure S6A) [7]. After focus is acquired, the slide can be removed from the stage and the prism and lens components can be assembled.

The lens is mounted to an XYZ linear stage which allows for fine adjustments to the focus and trajectory of the excitation beam (supplemental Figure S6B). To assemble the XYZ linear stage and lens, a 4” × 6” breadboard (Table 1, #56) is first mounted over the sI3–sL8 holes (Figure 2B) to elevate it off of the stage breadboard. The three micrometer actuators (Table 1, #58) are attached to the XYZ linear stage (Table 1, #57) and set to the center of their travel range (in this case ~6 mm). The lens (Table 1, #55) is then inserted into a fixed lens mount (Table 1, #14) such that the flat side will face the objective. A 2” post is attached to the fixed lens mount, a 3” post is attached to the top hole of the XYZ linear stage closest to the objective, and the two are connected using a 6” post and two right angle clamps (supplemental Figure S6B). The posts are then adjusted within the right-angle clamps such that the lens is approximately 5 cm from the objective and the beam is transmitted through the center of the lens and strikes the near edge of the objective’s front lens (supplemental Figure S6B—inset). When positioned properly, the beam should produce symmetrical points of light on opposite sides of the objective’s front lens.

The quartz prism is attached to a custom adaptor which allows the prism to be easily manipulated during operation of the microscope (see Section 4.1) (supplemental Figure S7A). During operation, the prism adapter is secured in place above the sample chamber by an overhead arm. Each time the sample chamber needs to be removed or replaced, the prism adapter and overhead arm must be removed first (see Section 4.1). Thus, the prism adapter and overhead arm are designed to be easily detachable from the stage. Similar to the stage breadboard and stage adapter pieces, the components used to support the prism (Table 1, #43–45) can be commissioned from a local machine shop using the design files provided on our lab website (https://www.freudenthallab.com/, accessed on 5 March 2021). To attach the prism to the prism adaptor, the prism connector piece (Table 1, #44) is first secured to the prism adapter (Table 1, #43) using the three prism adapter machine screws (Table 1, #49) (supplemental Figure S7A). A generic glass slide (Table 1, #77) is then placed on the stage and the prism adapter is secured to the overhead arm (Table 1, #45) via the central thumb screw (Table 1, #48) and mounted to the stage breadboard (supplemental Figure S7B). A Kimwipe is laid over the slide, and then the loose prism (Table 1, #73) is placed on the Kimwipe directly underneath the prism adapter. The prism can be repositioned as needed by gently sliding the Kimwipe. A thin coat of 5-min epoxy (Table 1, #74) is added to the top of the prism and then the prism adapter is released from the overhead arm and lowered until the connector piece makes contact with the top of the prism (supplemental Figure S7C). The prism adapter is then locked into place and the glue is allowed to set overnight. Attaching the prism in this way helps to ensure the prism is flat with respect to the slide while being secured to the overhead arm.

### 3.3. Emission Path

#### 3.3.1. Optosplit III Emission Splitting

The combined emissions from each fluorophore must be separated into three channels using dichroic mirrors before being projected onto their respective regions of the camera (Figure 1f,g). This is accomplished using an Optosplit III (Table 1, #65), which is connected to the left-side port of the inverted microscope (supplemental Figure S8A). To attach the Optosplit III to the microscope, the left side port cap is removed from the microscope by loosening the clamping screw. Then, the C-mount camera adapter (Table 1, #32) is secured to the entry port of the Optosplit III and inserted into left side port. The support foot of the Optosplit III is attached and adjusted such that the body of the Optosplit III is level with the plane of the table and the C-mount camera adapter is secured in place by tightening the left side port clamping screw. With the Optosplit III attached, the dichroic mirrors and emission filters used to partition the emission are mounted inside the filter cube and inserted into the Optosplit III housing as described in the user manual (Table 1, #59–63) (supplemental Figure S8B).

#### 3.3.2. EMCCD Camera

The final component of the prismTIRF microscope is the camera. In this setup, an electron multiplying charge-coupled device (EMCCD) (Table 1, #64) is used as the camera, although other comparable options are available [30,31]. To align the EMCCD with the exit port of the Optosplit III housing, the EMCCD camera is attached to a custom camera mount (Table 1, #46) (supplemental Figure S8A). To attach the camera mount, the two rubber stoppers on the side of the EMCCD camera are removed and two 1” long ¼”-20 screws (Table 1, #69) are used to secure the mount in place. The EMCCD camera can then be fixed onto the output port of the Optosplit III and the camera mount can be secured to the table using L-shaped clamps. If needed, L-shaped clamps can be used as risers to elevate the camera adapter off the table to align the camera to the Optosplit exit port (supplementary Figure S8A). If the EMCCD displays an image that is slanted, the two screws securing the camera to the camera mount can be loosened or tightened to fine-tune the camera orientation, which will rotate the depicted image. In the configuration shown here, the camera is turned 90° with respect to the Optosplit III, which allows for the USB and power cables to protrude from the side instead of down towards the table where there is little space. Although this will result in the image appearing to be rotated 90° clockwise, it can be easily corrected by rotating the image 90° counterclockwise in the camera settings and/or collection software settings.

After attaching the EMCCD to the Optosplit III, the EMCCD is connected to the computer via USB and can be operated using imaging software such as Micro-Manager (https://micro-manager.org, accessed on 5 March 2021), or the smFRET data acquisition and analysis package provided by Ha and colleagues (https://cplc.illinois.edu/research/tools, accessed on 5 March 2021) [22]. We use the software package provided by the Ha group, although the choice of software is primarily dependent on user preference and the file format requirements of the analysis software being used. Details regarding the configuration of the camera and operation of the software are available in the reference manual provided with the software package. After the EMCCD is attached, the adjustment knobs on the Optosplit III body can be used to adjust the position of each emission channel on the camera. Detailed information regarding the set up and operation of both the EMCCD and Optosplit III can be found in the manuals provided by the manufacturers.

## 4. Operation

### 4.1. Bead Slides and TIR Acquisition

A bead slide is a mock sample chamber which contains immobilized multi-fluorescent beads (Table 1, #76) (supplemental Figure S9) [7,14]. The bead slide is used to record a short mapping video which is used to calibrate the software that aligns the X–Y coordinates of the partitioned emission channel projections [3]. Additionally, the bead slide is useful for troubleshooting TIR acquisition. At times, it can be a challenge to find TIR on an experimental slide, especially if the position of the lens and prism are far from the proper position to induce optimal TIRF. However, the fluorescent beads within the bead slide are easy to see and will be visible even if the beam trajectory is near to, but not perfectly at, the proper angle to induce optimal TIRF. Therefore, it can sometimes be helpful to dial in TIR on a bead slide in order to get close to TIR before attempting to image experimental sample chambers.

Before setting up the microscope for TIRF imaging, the prism and slide are thoroughly cleaned using ethanol. This is critical, as even the smallest amount of debris, dried oil, and/or streaks can cause significant issues to the image and TIR quality. A drop of water is placed on the objective and the stage clips are used to secure the bead slide to the stage. The objective is then adjusted so that the seam of the tape or glue within the chamber is in focus in the eyepiece field of view (FOV) (supplemental Figure S6A). This will position the objective such that the TIR signal will be in focus once the prism and lens are properly positioned for TIRF imaging. It is important not to move the objective too far from this position while acquiring TIR, as it is difficult to focus the objective with the prism in place, and the TIR signal will not be visible if the slide/sample interface is out of focus. After the objective is positioned, the 532 nm beam is turned on and, if necessary, the lens micrometer is adjusted such that the beam strikes the left-most edge of the objective’s front lens (supplemental Figure S6C). Finally, the inverted microscope stage is moved using the short stalk stage handle (Table 1, #37) such that the center of the slide is directly over the objective and a drop of low-fluorescence immersion oil (Table 1, #78) is placed on the slide. The prism adapter and overhead arm are then mounted to the microscope stage and the prism is carefully lowered onto the drop of oil (supplemental Figure S10).

**Figure 4:**
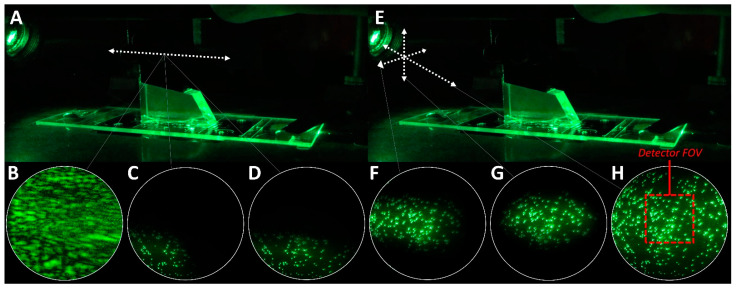
Acquiring TIR. (**A**) To acquire TIR at the imaged area of the slide, the 532 nm laser is turned on and the central thumb screw is loosened to release the prism adapter. The prism is then slid horizontally until the beam illuminates the region within the eyepiece field of view (FOV) (white circles). If the trajectory of the beam is correct, this should cause the hazy light (**B**) to be replaced with points of light on a dark background (**C**). (**D**) The prism is adjusted until the illuminated field is centered horizontally within the eyepiece FOV and resecured to the overhead arm via the central thumb screw. (**E**) Afterwards, the lens micrometers are used to make fine adjustments to the beam trajectory and focus. (**F**–**G**) The micrometers are adjusted to center the illuminated field within the eyepiece FOV. (**H**) The illuminated field can be narrowed or expanded to fill the eyepiece FOV by moving the lens towards or away from the prism along the beam path, respectively. This is carried out by incrementally adjusting the lens down and toward the prism, or up and away from the prism, while also looking through the eyepiece to ensure the illuminated field remains in view. The camera FOV (red box) is only a small central region of the eyepiece FOV.

Acquiring optimal TIR for TIRF imaging involves tuning the trajectory and focus of the excitation beam by making fine adjustments to the positioning of the prism and lens (Figure 4). Acquiring TIR begins with sliding the prism horizontally until the beam illuminates the region of the slide within the eyepiece FOV (Figure 3B). If the trajectory of the beam is correct, a dark background with a field of points of light will emerge in the eyepiece FOV (Figure 4B–D) and the reflected beam should produce a symmetric circle on the far wall (Figure 3A). If this cannot be obtained, double-check that the objective is set to the correct height and that the beam strikes the front edge of the objective (supplemental Figure S6). After making adjustments to the prism, the illuminated field can be centered (Figure 4H) and expanded or narrowed to fill the eyepiece FOV (Figure 4E–G) by adjusting the position of the lens via the lens micrometers (Figure 4E–H). The camera observes only a small central region of the image seen through the eyepiece (Figure 4H, red box). Thus, expanding the illuminated field to fill the eyepiece FOV ensures that the camera FOV contains homogenously excited fluorophores. It is recommended to turn off all lasers except the 532 nm laser when acquiring TIR, since the other beams will be aligned with respect to the 532 nm beam later on (see Section 4.2).

### 4.2. Excitation Beam Alignment

**Figure 5:**
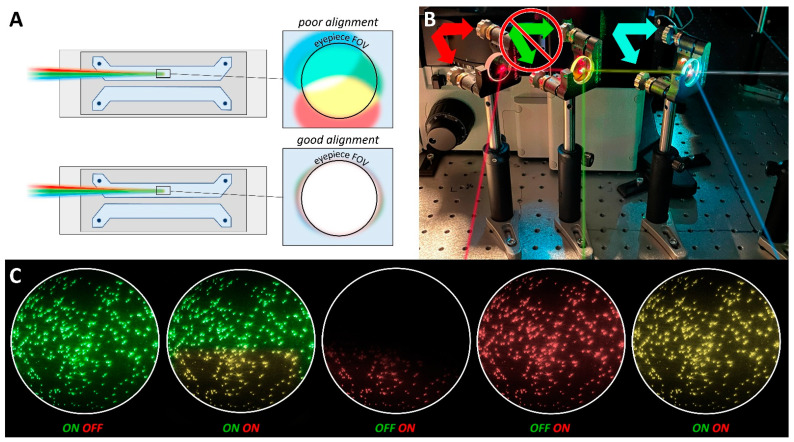
Aligning the excitation beams. (**A**) The 640 nm and 488 nm beams require alignment if their illuminated fields do not overlap with the centered 532 nm beam. (**B**) The 640 nm and 488 nm beams can be aligned independently by tuning the adjustment knobs on the broadband mirror at Y43 and the 505 nm cut-off dichroic mirror at R43, respectively. Importantly, the 550 nm cut-off dichroic mirror should not be adjusted, as this will forfeit the original beam path established during construction. (**C**) The adjustment knobs of the mirror mounts are tuned until the illuminated fields are centered within the eyepiece FOV. When the 640 nm and 488 nm beams are properly aligned, the color of the illuminated field will be homogenous when all lasers are on.

After the region illuminated by the 532 nm beam is adjusted to fill the eyepiece FOV, the other beams (488 nm and 640 nm) may need to be aligned so that they illuminate the same field as the 532 nm beam (Figure 5A). If the 488 nm and/or 640 nm beams are out of alignment, the illuminated field will appear off-center within the eyepiece FOV. To correct this, the trajectory of the 488 nm and 640 nm beams can be independently adjusted by turning the adjustment knobs on the 505 nm dichroic mirror and broadband mirror that are used to merge the excitation beams, respectively (Figure 5B). When the beams are aligned, the color of the illuminated beads should be homogenous over the eyepiece FOV when all lasers are on (Figure 5C). Importantly, the trajectory of the 532 nm beam should never be adjusted by turning the adjustment knobs on the 550 nm dichroic mirror, as doing so will cause the path of the merged excitation beam that was established during construction to be lost. The 532 nm beam should only be adjusted using the prism and/or lens micrometers.

### 4.3. Emission Channels and Camera Settings

The emissions are visualized and recorded using the EMCCD, a computer, and an imaging software such as the Single.exe program contained within the smFRET data acquisition and analysis package provided by the Ha group [22]. Here, we provide a brief overview of the operation of the camera and the Single.exe imaging software. More detailed information can be found on the EMCCD manufacturer’s website and in the Single.exe reference manual [22]. To visualize the emission channels, the power button on the back of the EMCCD is turned on and the Single.exe program is opened. The camera is cooled to −80 °C and the PMA recorder window is opened. After confirming that the illuminated field is centered within the eyepiece and the excitation beams are aligned (Figure 5C), the light-path is diverted completely to the camera using the light path selector lever on the right side of the inverted microscope. Finally, before activating the camera, the lights in the room are turned off. When imaging a bead slide with Single.exe, the gain should be reduced to ~10 and the “Background” and “Data Scaler” settings should be set to “Autoscale”. The 532 nm beam is turned on and, if needed, the laser power should be adjusted such that the bead spots depicted in the imaging software are blue with a slight red center. Bright white spots will saturate the camera and result in an unusable mapping file. Note that fine adjustments to the focus of the inverted microscope may be required to sharpen the image. When imaging a sample chamber, the gain should be increased to 299 and the “Background” and “Data Scaler” settings should be adjusted to acquire a clear and well contrasted image. Finally, to maintain consistency between data sets, it is recommended to keep these settings constant between experiments within a dataset.

The position of each emission channel on the camera can be adjusted using the adjustment knobs on the top and side of the Optosplit III (supplemental Figure S8A). Detailed information regarding this procedure is outlined in the Optosplit III manual. If three emission channels are to be recorded simultaneously, the size of the channels can be adjusted to each fill a third of the total detector area using the aperture control levers located on the entry port of the Optosplit III (supplemental Figure S8A). If only two emission channels are needed for an experiment, a blocker can be easily placed into the Optosplit III housing to block the path of the unnecessary channel, and the other channels can be adjusted to each fill half of the detector space. The position of each channel should be adjusted similar to the depiction shown in Figure 1, with space around the edges of each channel so they do not overlap or extend beyond the edges of the detector.

At the start of each week of experiments, or after making any adjustments to the position of the emission channels, a new mapping file should be recorded. A mapping file is a short 100 frame video of the stationary beads within a bead slide that is used to map the X–Y coordinates of each emission channel [3]. During processing of an experimental data video, the mapping file will be used to define the region of each emission channel so they can be overlaid. This process is explained in detail in the documentation provided with the smFRET data acquisition and analysis package [22].

### 4.4. Sample Chamber Assembly

**Figure 6:**
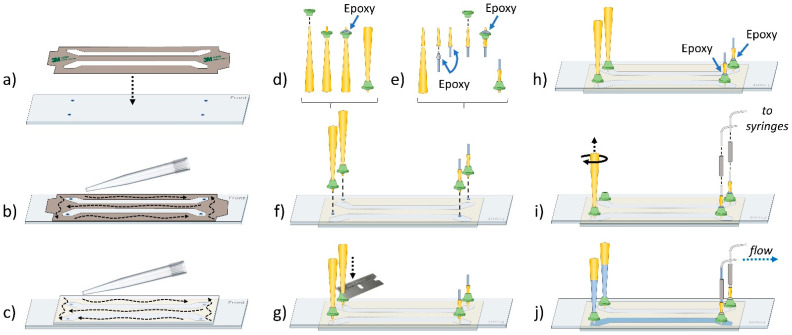
Building sample chambers. (**a**) A sheet of double-sided adhesive is cut to create flow chambers between the drilled holes of the quartz slide. The quartz slide is placed on a clean surface PEG-side up. The backing on one side of the adhesive is removed and the adhesive is positioned onto the slide. (**b**) A pipette tip is pressed against the back of the adhesive to adhere it to the slide. (**c**) The second backing of the adhesive is removed, and a coverslip is placed over the adhesive, PEG-side down. A razor is used to remove any excess adhesive surrounding the coverslip and a pipette tip is used to press on the coverslip to secure the bond. The sample chamber is then flipped over so the holes are exposed. (**d**–**g**) The entry and exit ports are installed one at a time. (**d**) Entry ports are installed by sliding a 3D printed port onto the end of a 200 µL tip (Table 1, #81). A smear of 5-min epoxy is applied to the bottom of the port carefully to avoid getting glue on the very end of the tip. (**f**) The tip is inverted and inserted into a hole on the sample chamber. (**g**) While using a finger to put pressure on the top of the tip to keep it in the hole, the side-notch of a razor blade is used to push the port down firmly to the slide surface. This is repeated to install the second entry port. (**e**) To install an exit port, the top ¾” is severed from a 200 µL tip using a razor blade. A ring of glue is applied to the outside of one end of a 1” long piece of ETFE tubing (Table 1, #80) and inserted firmly into the shortened tip. Batches of these tip/tubing assemblies are made in advance to save time. Exit ports are then installed following the same procedure as the entry ports (**e**–**g**). (**h**) Epoxy is applied to the top of each exit port to ensure that any tugging of the connected tubing does not dislodge the tip. The 5-min epoxy is allowed to dry for at least 20 min, then any excess glue or smudges are removed using Kimwipes and a small amount of acetone and/or ethanol. (**i**) Two 1” lengths of flexible tubing are used as connectors to link the tubing of the exit ports to the tubing leading to the syringes. The 200 µL tips in the entry ports are dislodged from the glue with a firm twist and removed. (**j**) New 200 µL tips containing solution can be inserted into the entry ports and the solution can be drawn into the sample chamber by pulling suction on the syringe connected to the exit port.

The experimental sample chamber contains the fluorescent sample(s) under observation. It consists of a quartz slide (Table 1, #75) with two or more holes drilled into its surface, a glass coverslip, and an adhesive to attach the two together and create a channel between the holes (Figure 6) [32]. The quartz slide and coverslip require extensive cleaning and passivation to minimize background and noise, and detailed information regarding this process is readily available [33,34,35]. After the surfaces are prepared, sample chambers have traditionally been constructed using double stick tape to create flow channels between the holes in the slide and epoxy to seal the ends [33]. Although double stick tape is effective and is still used successfully in many labs, we have found that using adhesive sheets (Table 1, #83) cut with either a razor blade or a cutting machine (e.g., Cricut) to build the flow channels prevents the need of 5-min epoxy to seal the ends of the channel and significantly reduces the time and technicality of building sample chambers (Figure 6a–c). The cutting machine designs we use can be found on our lab website (https://www.freudenthallab.com/, accessed on 5 March 2021).

In many cases, it is desired to load solutions into the sample chamber without removing the prism. Traditionally, this issue has been solved by gluing 200 µL tips and tubing directly into the holes of the slide [6]. However, we have found the tips can often come loose with this method, and if multiple samples are to be run through the same chamber, the repeated use of the entry tubing can cause contamination between samples. With this in mind, we have designed 3D printable ports which can be printed using a relatively cheap 3D printer (e.g., Anycubic Photon). The ports can be glued to the slide and support the insertion of a 200 µL pipette tip (Table 1, #81) directly into the holes of the sample chamber (Figure 6d–i). The port on one end of the chamber is used to permanently secure a make-shift adapter into the hole of the slide, which is connected to syringes via FPLC tubing. The opposite port is used to support a 200 µL pipette tip which serves as a replaceable reservoir for solutions that can be drawn into the chamber via suction from the syringes (Figure 6j) (supplemental Figure S11C). The benefit of this is that the incoming sample does not come into contact with tubing or other surfaces that might contain contaminates. The reservoir can be topped off by pipetting directly into the top of the existing reservoir tip and pulling the solution into the chamber using the syringe (supplemental Figure S11A). Additionally, replacing the reservoir tip is as easy as drawing the solution into a fresh tip with a pipettor, inserting the new tip into the port, and ejecting the tip from the pipettor (supplemental Figure S11B). Overall, these ports allow for multiple experiments to be conducted on a slide without the need to disassemble the prism, allow for multiple solutions to be flowed through the chamber during recording of a video without concern of sample contamination, and prevent the need to clean or replace tubing and syringes between experiments. The files necessary to print these ports are provided on our lab website (https://www.freudenthallab.com/, accessed on 5 March 2021).

### 4.5. Data Collection

The three-color prismTIRF microscope described here is capable of simultaneously imaging three different fluorescent molecules. Therefore, it can be used to study complex assembly kinetics involving up to three fluorescently labeled components. For example, to study DNA binding proteins which form co-complexes on DNA, one can immobilize fluorescently labeled biotin-DNA oligos (e.g., AF488) to the slide and then flow two different fluorescently tagged DNA binding proteins (e.g., Cy3 and Cy5) into the chamber (supplemental Figure S12A). To carry out this experiment, a bead slide is first used to set the objective the appropriate height (Section 4.1), the prism adapter and overhead arm are attached to the stage over the bead slide (supplemental Figure S10), the 532 nm laser is turned on, TIRF is centered in the eyepiece FOV (Section 4.1), and if need be the lasers are aligned (Section 4.2). A mapping file of the bead slide is recorded (Section 4.3) and then the overhead arm, prism adapter, and bead slide are removed and cleaned thoroughly with ethanol. A sample chamber is assembled (Figure 6) and mounted to the stage such that the channel of the sample chamber is directly over the objective. The exit ports are connected to syringes and a 200 µL tip containing buffer (e.g., HEPES 50 mM, NaCl 150 mM) is inserted into the entry port and ejected from the pipettor (supplemental Figure S11B). The exit port syringe is then used to draw the buffer through the chamber carefully, to avoiding pulling air into the sample. Additional buffer is added to the top of the reservoir and pulled through until ~600 µL of buffer has been flushed through the chamber.

To immobilize the biotin-DNA to the biotin-PEG coating of the slide, ~150 µL of 0.2 mg/mL neutravidin is pipetted into the reservoir and pulled into the chamber. After 3 min, the neutravidin solution is flushed out with ~600 µL of buffer. Before adding any fluorescent molecules to the sample chamber, the overhead arm and prism adapter are mounted to the stage and TIRF is once again centered in the eyepiece FOV. At this point, we find that pre-bleaching the slide by turning on all lasers for ~5–10 min minimizes noise and non-specific fluorescence that can occur during data collection. Afterwards, the lasers are turned off and the fluorescently labeled biotin-DNA is flowed into the chamber and incubated for 3 min. We find 10–100 pM biotin-DNA works well in our system, although the optimal concentration must be determined empirically. Furthermore, it is imperative that any solutions containing fluorophores (imaging buffer) should include oxygen scavenging reagents [32]. After 3 min, the DNA solution is flushed out with ~200 µL of imaging buffer. Finally, the fluorescently labeled proteins are flowed into the chamber, the lasers are turned on, and the recording can be started. Similar to the biotin-DNA, the optimal concentrations of proteins will need to be empirically determined and can range dramatically based on the experimental conditions (0.01–10.0 nM).

When the experimental video has finished recording, the video is processed using an environment such as IDL, which can operate scripts to convert the video into a collection of trajectories [22,32]. Each trajectory displays the fluorescence intensity of each emission channel observed at an immobilized molecule over the course of the video (supplemental Figure S12B). Finally, the individual binding events seen in the trajectories are quantified, and a statistical approach is taken to analyze the various populations of event types and durations (supplemental Figure S12C) [9]. This can be accomplished using analysis tools such as the kinetic event resolving algorithm (KERA) provided by the Spies group (https://github.com/MSpiesLab/KERA, accessed on 5 March 2021), which can organize and sort the fluorescent trajectories from a range of single-molecule experiments [9,36]. The dissociation and association rate kinetics between each protein and the DNA can then be determined through statistical analysis of the binary binding event durations and the time between binding events [9,37]. Additionally, ternary events where both proteins bind to the DNA simultaneously can be sorted and analyzed to study the intricacies of complex assembly [9,38].

## 5. Applications

One of the primary reasons that single-molecule TIRF microscopy is becoming increasingly popular is due to its wide variety of applications, including the ability to study macromolecular complexes. The build described above is for a three-color TIRF microscope, which offers some advantages in comparison to one- and two-colored systems. Specifically, three-color TIRF microscopes allow for more experimental flexibility as the three fluorophore markers allow additional components of multicomponent macromolecular complexes to be simultaneously visualized (supplemental Figure S12A). For example, in the case of protein–DNA complexes, this could include multiple labels on DNA to monitor DNA structural dynamics in the presence of a labeled protein or up to three labeled proteins on unlabeled DNA. Moreover, users could perform three different combinations of dual fluorophore experiments.

Here, we briefly review select examples of how TIRF microscopy has been applied to the study of complex DNA structures and multi-component protein–DNA complexes responsible for homologous recombination, DNA replication, DNA repair, and transcription. Due to space constraints, we were limited in the number of investigators we could highlight in this section and apologize to those who have contributed to the development and application of single-molecule assays but were not mentioned herein. Specific techniques that utilize TIRF microscopes to study protein–DNA complexes include DNA or protein tethering [39,40,41,42], DNA combing and curtains [43,44,45,46], DNA tightropes (via oblique angle fluorescence imaging) [47,48], as well as smFRET [40,49,50,51,52,53] and TIRF/AFM systems [54,55,56]. While the examples mentioned below include data collected on both prism and objective-based TIRF instruments, in most cases, the prismTIRF microscope described above can be used for similarly designed experiments.

Much of the development and application of single-molecule techniques suitable for the study of nucleic acid systems was pioneered by the lab of Taekjip Ha [57,58,59,60,61,62,63,64,65,66,67]. This includes the first demonstration of a protein diffusing on single-stranded DNA (ssDNA) [59], combining force and fluorescence to probe Holliday junction dynamics [58], exploring the activities of various DNA repair helicases [39,61,62,64,65], DNA strand exchange [66,67], and nucleosome dynamics [63] to name a few. Importantly, this work laid the foundation for many other groups to further the development of single-molecule techniques and explore other nucleic acid systems including recombination, DNA replication, DNA repair, and transcription.

Homologous recombination (HR) is an important mechanism for the repair of double-strand breaks (DSBs) and has been studied extensively via single-molecule techniques [43,68,69,70,71,72,73,74,75,76]. This includes the ssDNA curtain assay developed by the Greene lab, which has provided new insight into HR regulation and the complex and heterogenous reaction intermediates that are involved [43,76]. One example is the multi-functional Mre11/Rad50/Nbs1 (MRN) complex, which initiates DSB repair by recognizing and resecting the free DNA ends at damaged sites. The Finkelstein lab used DNA curtains to investigate how the MRN complex initiates DNA resection, assembles the larger resectosome, and is regulated by DNA-dependent protein kinase [68,69,70]. Another aspect of HR that has been studied using a TIRF microscope is the assembly of Rad51 filaments along ssDNA. Utilizing Protein-Induced Fluorescence Enhancement (PIFE), which utilizes a fluorophore attached to the substrate as a reporter of protein binding and dynamics [72], the Sua Myong lab monitored Rad51 filament formation on fluorescently labeled ssDNA substrates [73]. Interestingly, these studies suggest that filament formation occurs in the 5’ to 3’ direction and that the anti-recombinase Srs2 acts to transiently counterbalance Rad51 filament formation. Moreover, the Spies lab used TIRF microscopes to follow the formation kinetics of filament nuclei at a single-monomer resolution, observing that human Rad51 nucleates and grows in dimers [74], which is in contrast to the addition/dissociation of monomers of *E. coli* RecA [75] and yeast Rad51 [73].

DNA replication requires several multi-factor components (i.e., a helicase, multiple DNA polymerases, a primase, and a ring-shaped sliding clamp protein). Due to the rapid and uneven fashion by which replisomes travel on DNA, several key aspects of their behavior are undetectable by ensemble studies. This has led multiple labs to use single-molecule fluorescence techniques to study the replisome [77,78,79,80,81,82,83,84,85]. For example, the van Oijen group revealed new insight into the coordination of the bacteriophage T7 replisome by simultaneously monitoring the kinetics of DNA loop growth in the lagging strand and leading strand synthesis [79]. Around the same time, the Kowalczykowski lab used single-molecule analysis to determine that the leading and lagging strand polymerases function autonomously within a replisome and that replication is kinetically discontinuous and punctuated by pauses and rate-switches [80]. Additionally, the O’Donnell group was able to measure the processivity and rate of replisome activity in real time using a single-molecule TIRF rolling circle replication assay, revealing that the lagging strand increases replisome processivity but slows replication fork progression [77]. Single-molecule TIRF has also been used to investigate how the structurally isolated *pol* and *5’-nuc* DNA binding domains of DNA polymerase I (Pol I) are utilized during interactions with DNA. Using smFRET, the Millar group identified separate subpopulations of DNA engaging the two DNA binding domains and found the relative populations and dwell times of each complex varied according to the nature of the DNA substrate [78]. Interestingly, additional experiments showed that DNA substrates can switch between the *pol* and *5’ nuc* DNA binding domains during a single encounter with Pol I.

DNA repair is usually accomplished by large multi-component repair complexes. One such component is Replication Protein A (RPA), a ssDNA binding protein which is required for many DNA repair pathways, including nucleotide excision repair (NER), recombinational repair (HR and MMHJ), and mismatch repair (MMR) [41]. The Wold group applied single-molecule TIRF microscopy to analyze binding between DNA and surface-tethered RPA, revealing that RPA–DNA interactions can form two distinct complexes which are both critical for RPA function [41]. Single-molecule studies have also shown that RPA can resolve noncanonical G-quadruplex DNA structures which can interfere with DNA repair [86,87]. To study another DNA repair factor, XPD helicase, the Spies group reported a single-molecule imaging strategy that utilized the FeS-mediated quenching of a site-specific fluorophore, which allowed for real-time and direct correlation between nanometer-scale domain motions and its interaction with individual DNA substrates [10]. Additionally, using single-molecule TIRF, the Washington laboratory examined the assembly and disassembly of the multi-protein complex that carries out translesion synthesis (TLS) [38]. They found that ternary complexes containing proliferating cell nuclear antigen (PCNA) and two specialized TLS DNA polymerases, Rev1 and DNA polymerase η, have two architectures: PCNA tool belts and Rev1 bridges. Moreover, these complexes were shown to be dynamic and their architectures can interconvert without dissociation, possibly facilitating the selection of the appropriate polymerase and polymerase-switching events during TLS.

Chromatin fibers are a key determinant of genome regulation as it dictates the accessibility of DNA to proteins. Single-molecule techniques have been instrumental in understanding protein–chromatin interactions [88,89,90,91,92]. For example, single-molecule analysis has demonstrated that the nucleosome remodeler SWR1 destabilizes the DNA wrapped around the histone core, and this partial unwrapping is regulated by ATP binding [91]. The Fierz group used smFRET to directionally map local chromatin structural states and measure their interconversion dynamics [89]. In addition, by moving the fluorescent labels to several positions, structural information was obtained from several vantage points. This revealed that nucleosomes engage in effector protein-regulated stacking interactions, which rapidly interchange on a timescale of micro-to-milliseconds. An additional study by the Lenstra lab combined in vitro and in vivo single-molecule imaging approaches, allowing them to observe the direct correlation between binding of the Gal4 transcription factor and the transcriptional bursting kinetics of the Gal4 target genes GAL3 and GAL10 in living yeast cells [88]. Interestingly, they found that the Gal4 dwell time sets the transcriptional burst size, and that the Gal4 dwell time is reduced by nucleosomes.

## Figures and Tables

**Table 1:** Component list.

Ref #	Item	Notes	Catalog ID	Vendor	#
	Table				
**#1**	Optical Table	3’ × 6’ × 12”, 1/4”-20 threads	RPR-36-12	Newport	1
**#2**	Table Legs	SL-600 Series, 22” height, 2400 lb max. capacity	SL-600-422	Newport	4
**#3**	Overhead Table Shelf	Fits 6’ table, w/ electrical outlets	ATS-6	Newport	1
	Mounting Hardware, Clamps, and Posts				
**#4**	2" Post	1/2” diameter, top tapped 8-32, bottom tapped 1/4”-20	9621	Newport	4
**#5**	3” Post	1/2” diameter, top tapped 8-32, bottom tapped 1/4”-20	9622	Newport	1
**#6**	4” Post	1/2” diameter, top tapped 8-32, bottom tapped 1/4”-20	9623	Newport	7
**#7**	6” Post	1/2” diameter, top tapped 8-32, bottom tapped 1/4”-20	9624	Newport	20
**#8**	4” Adjustable Post Holder	Fits 1/2” diameter posts, bottom tapped 1/4”-20	VPH-4PK	Newport	20
**#9**	Pedestal Base Adaptor	1-1/4” diameter, 0.19” height, top thread 1/4”-20	PS-A-PK	Newport	20
**#10**	Slotted Clamping Fork	Fits Pedestal Base Adaptor (#9), slot for 1/4”-20 bolt	PS-F-PK	Newport	30
**#11**	L-shaped Table Clamp	(for mounting #20, #30, #46)	CL5-P5	Thorlabs	19
**#12**	Right-Angle Post Clamp	Fits 1/2” diameter posts (#4–7)	9935	Newport	7
**#13**	1” Ultima Clear Edge Mirror Mount	Fits 1” diameter mirrors (#27–#29), 2 knobs, left-handed	U100-A-LH-2K	Newport	13
**#14**	1/2” Lens Mount	Fits 1/2” diameter lenses (#24-26, #55), 8–32 Thread	LH-0.5A	Newport	4
**#15**	1” Adjustable Post Holder + Pedestal Base	Fits 1/2” diameter posts (for stage mirror assembly)	VPH-1-P	Newport	1
	Excitation Lasers				
**#16**	OBIS 488nm LS 100 mW Laser	CW, Diode, beam diameter 0.7 ± 0.05 mm, (to excite AF488)	1226419	Coherent	1
**#17**	OBIS 532nm LS 100 mW Laser	CW, Diode, beam diameter 0.7 ± 0.05 mm, (to excite Cy3)	1261781	Coherent	1
**#18**	OBIS 640nm LX 100 mW Laser	CW, Diode, beam diameter 0.8 ± 0.1 mm, (to excite Cy5)	1185055	Coherent	1
**#19**	OBIS Laser Heat Sink	Fits LX/LS OBIS Lasers, 2.7” height (optional)	1193289	Coherent	3
**#20**	Vertical Translation Stage	84 mm–184 mm height, M6 thread holes (mounted via #11)	860-0075	Eksma	3
**#21**	OBIS Laser Remote and Power Supply	Includes six 1-meter SDR cables	1234466	Coherent	1
	Laser Shutters				
**#22**	Laser Shutter	3mm laser shutter, teflon coating, no electronic sync	LS3S2T0-100	Uniblitz	3
**#23**	Shutter Driver	four-channels, includes 710C shutter interconnect cables	VMM-D4	Uniblitz	1
	Excitation Dichroic Mirrors and Filters				
**#24**	488 nm Laser clean-up filter	0.5” diameter, 488 nm, MaxLine®	LL01-488-12.5	Semrock	1
**#25**	532 nm Laser clean-up filter	0.5” diameter, 532 nm, MaxLine®	LL01-532-12.5	Semrock	1
**#26**	640 nm Laser clean-up filter	0.5” diameter, 640/8 nm, MaxDiode™	LD01-640/8-12.5	Semrock	1
**#27**	Broadband Mirror	1” diameter, 6.0 mm thick, RWE: λ/10 @ 633 nm	BB1-E02	Thorlabs	11
**#28**	505 nm Cut-off Longpass Dichroic Mirror	1” diameter, 3.2 mm thick, TWE: λ/4 @ 633 nm	DMLP505	Thorlabs	1
**#29**	550 nm Cut-off Longpass Dichroic Mirror	1” diameter, 3.2 mm thick, TWE: λ/4 @ 633 nm	DMLP550	Thorlabs	1
	Inverted Microscope Components				
**#30**	IX73 Microscope Frame (1 deck)	Single deck	IX73P1F	Olympus	1
**#31**	60X Water Objective (NA 1.20)	UPLSAPO60XW; U Plan S-Apo, WD0.28,W/CC0.13-0.21	1-U2B893	Olympus	1
**#32**	C-Mount Camera Adapter	Centerable (U-TV1XC) (1x Mag)	U-V111C	Olympus	1
**#33**	Fluorescent Turret	(IX3-RFACS-1-2 CODED)	5-UR416-1	Olympus	1
**#34**	Binocular Observation Tube	GX/IX (U-BI90-1-2)	3-U243	Olympus	1
**#35**	10X Eyepiece	(FN:22 WHN10X-1-7)	2-U1007	Olympus	1
**#36**	10X Eyepiece (Adjustable Focus)	(FN:22 WHN10X-H-1-7)	2-U100H6	Olympus	1
**#37**	Left Handle Stage with Short Stalk	(IX3-SVL)	4-U222	Olympus	1
**#38**	Stage Clips	FOR IX STAGE (IX-SCL) (includes 2)	FV4-U291	Olympus	1
**#39**	6-position Nosepiece	(IX3-D6RES CODED)	U-R380	Olympus	1
	Custom Components and Screws				
**#40**	Custom Stage Adapter (Rear)	Schematics can be found at www.freudenthallab.com (accessed on 21 June 2021)	CAD file ID 40	machine shop	1
**#41**	Custom Stage Adapter (Front)		CAD file ID 41	local shop	1
**#42**	Custom Stage Breadboard		CAD file ID 42	local shop	1
**#43**	Custom Prism Adapter		CAD file ID 43	local shop	1
**#44**	Custom Prism Connector Piece		CAD file ID 44	local shop	1
**#45**	Custom Prism Overhead Arm		CAD file ID 45	local shop	1
**#46**	Custom Camera Mount		CAD file ID 46 (A–C)	local shop	1
**#47**	Custom Slide Holder Insert		CAD file ID 47	local shop	1
**#48**	Thumb Screws	1/4”-20 × 3/4”, knurled head 3/4” diameter	60746807	mscdirect	3
**#49**	Prism Connector Screws	4-40 × 1/4”, Mini Socket Cap Screw	43494	Hillman	3
**#50**	Camera Mount Screws	10-32 × 3/4”, Zinc, Flat Head, Phillips	101100	Hillman	4
**#51**	Front Stage Breadboard Screws	1/4”-20 × 1-1/4”, Zinc, Flat Head, Phillips	101140	Hillman	2
**#52**	Front Stage Breadboard Screws	M5—0.8 mm × 40 mm, Socket Cap Screw	43103	Hillman	2
**#53**	Back Stage Breadboard Screws	10-24 × 1”, Socket Cap Screw	3197	Hillman	2
**#54**	Back Stage Breadboard Screws	M5—0.8 mm × 25 mm, Socket Cap Screw	43102	Hillman	4
	Focusing Lens Micrometer Components				
**#55**	Plano-Convex Lens	1/2” diameter, 50.0 mm focal length, uncoated	LA1213-N-BK7	Thorlabs	1
**#56**	Optical Breadboard (4” x 6”)	1/4”-20 thread on 1” grid, aluminum	SA2-04x06	Newport	1
**#57**	XYZ Quick-Mount Linear Stage	1/2” travel, right-handed, 1/4”-20 thread	460A-XYZ	Newport	1
**#58**	Micrometer Actuator	1 µm vernier, 13 mm travel, 50.8 TPI (fits #57)	SM-13	Newport	3
	Emission Dichroic Mirrors and Filters				
**#59**	AF488 Bandpass Filter	1” diameter, 512/25, BrightLine®	FF01-512/25-25	Semrock	1
**#60**	Cy3 Bandpass Filter	1” diameter, 585/40, BrightLine®	FF01-585/40-25	Semrock	1
**#61**	Cy5 Bandpass Filter	1” diameter, 680/42, BrightLine®	FF01-680/42-25	Semrock	1
**#62**	532 nm Cutoff Longpass Dichroic Mirror	25.2 × 35.6 × 1.1mm, RWE: 1λ P-V @ 632.8 nm, BrightLine®	DI03-R532-T1-25X36	Semrock	1
**#63**	635 nm Cutoff Longpass Dichroic Mirror	25.2 × 35.6 × 1.1mm, RWE: 1λ P-V @ 632.8 nm, BrightLine®	DI03-R635-T1-25X36	Semrock	1
	Camera and Emission Splitter				
**#64**	IXON ULTRA 897 EMCCD	56 FPS, 512 X 512, 16 UM, USB	OAT-DU-897U-CS0-#BV	Olympus	1
**#65**	Optosplit III	Three-way image splitter,LS, 1X mag (contains #59–63)	O89-P280/310/0LS	Cairn	1
	Tools and Screws				
**#66**	Hex Driver Set (Imperial)	20-Piece Balldriver and Hex Key Kit, w/Stand, Imperial	TC2	Thorlabs	1
**#67**	Hex Driver Set (Metric)	15-Piece Balldriver and Hex Key Kit, w/Stand, Metric	TC3/M	Thorlabs	1
**#68**	1/2” Spanner Wrench	For threaded retaining rings (for 1/2” fixed lens mounts)	LT05-WR	Newport	1
**#69**	Imperial Screw and Hardware Kit (1/4”-20)	1/4”-20 Cap Screw and Hardware Kit	HW-KIT2	Thorlabs	1
**#70**	1” Iris	25.0 mm max aperture, TR3 Post (used for alignment)	ID25	Thorlabs	2
**#71**	Thin Slip-On Post Collar	Fits 0.5” diameter posts (maintains height of iris)	R2T	Thorlabs	2
**#72**	Beam Height Measurement Tool	12” tall, (used to measure height of beam and iris)	BHM4	Thorlabs	1
	Miscelaneous/Consumables				
**#73**	Pellin-Broca Quartz Prism	Fused Silica	325-1206	Eksma	1+
**#74**	5 Minute Epoxy	Devcon, syringe	12084	Tap Plastics	1+
**#75**	Quartz Microscope slide	1” × 3” × 1 mm	1X3X1MM	Finkenbeiner	1+
**#76**	TetraSpeck Microspheres	0.2 µm diameter, blue/green/orange/dark red	T7280	Thermo Fisher	1+
**#77**	Plain Glass Microscope Slides	Glass, 25 × 75 mm, 90° Ground Edges, Plain	1301	Globe Scientific	1+
**#78**	Low Autofluorescence Immersion Oil	n = 1.518, Olympus Type F, 30 mL	MOIL-30	Thorlabs	1+
**#79**	Solvent Dropper Bottle	2 oz. (60 mL) (to add water to objective)	LAB-14	Newport	1
**#80**	ETFE Tubing (ID 1.0 mm, OD 1/16”)	3 m length, translucent	18114238	Cytiva	1+
**#81**	Fisherbrand™ Redi-Tip™ 200 µL Pipet Tips	General purpose, yellow, 1000/PK	02-707-500	Fisher Scientific	1+
**#82**	HEPA Air Purifier	Holmes small room 3-speed, w/optional ionizer	B0000DK35B	Holmes	1
**#83**	3M Double-Sided Adhesive Sheet	Clear, 5 MIL, double linered, 12 × 12”	7955MP	Hisco	1+
**#84**	Double Stick Tape	Scotch 665 Permanent 3M, Permanent, 1/2” × 250”, Clear	917243	Office Depot	1+
**#85**	Cover Glass	24 × 60 mm, No 1.5, 1 oz	48393-251	VWR	1+

## Data Availability

Not applicable.

## Supplementary Materials

The following are available online at https://www.mdpi.com/article/10.3390/biology10070571/s1, Figure S1: Lasers, shutters, and clean-up filters. Figure S2. Leveling the excitation beams. Figure S3. Beam path to microscope stage. Figure S4. Mounting the stage breadboard. Figure S5. Stage mirror and TIRF angle. Figure S6. Plano-convex lens and XYZ linear stage assembly. Figure S7. Attaching the prism to the prism adapter. Figure S8. Emission path. Figure S9. Bead slide assembly. Figure S10. Mounting the prism adapter and overhead arm. Figure S11. Using a sample chamber. Figure S12. Representative Data.
