# Supplementary material for: Construction of a Three-Color Prism-Based TIRF Microscope to Study the Interactions and Dynamics of Macromolecules
Source: Biology (Basel). 2021 Jun 23;10(7):571. doi: 10.3390/biology10070571 (PMC8301196; doi:10.3390/biology10070571)
Supplement: Supplementary file 1 [file biology-10-00571-s001.zip › biology-1236469-supplementary.pdf]

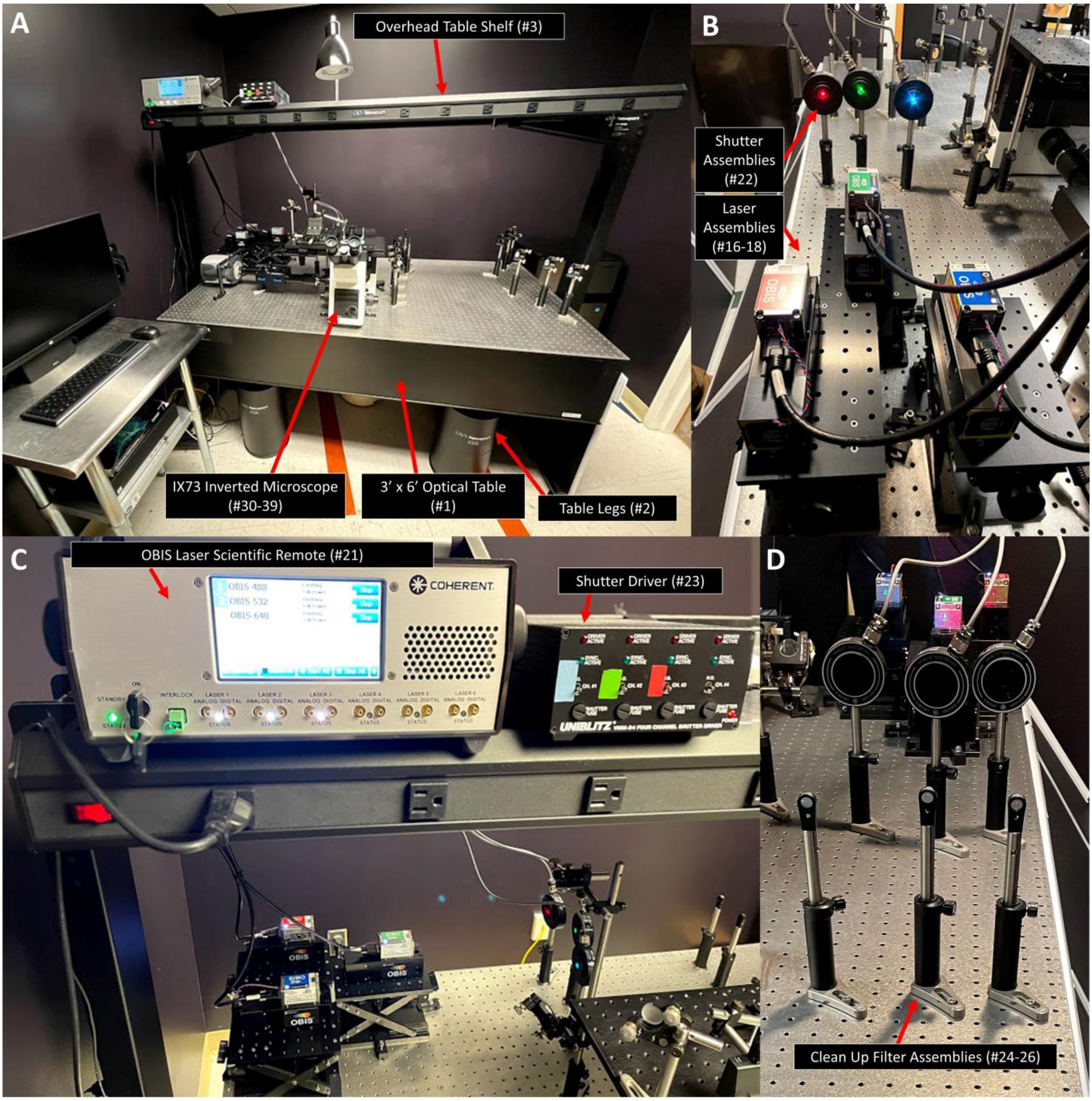

Supplemental Figure 1. Lasers, shutters, and clean-up filters. (A) The prismTIRF microscope components are assembled on an optical table which is supported by four pneumatic legs. An overhead shelf is used to store the OBIS laser scientific remote and shutter driver. (B) Each laser head is mounted to a heat sink, secured to a vertical translation stage, and attached to the table using three L-shaped clamps. Each shutter is attached to a 4" post, an adjustable post holder, a pedestal base adaptor, and secured to the table with a slotted clamping fork. (C) The OBIS laser scientific remote and shutter driver are positioned on the overhead shelf within reach of the operator. (D) Each laser clean-up filter is mounted inside a fixed lens mount and attached to a 6" post, an adjustable post holder, a pedestal base adaptor, and secured to the table with a slotted clamping fork.

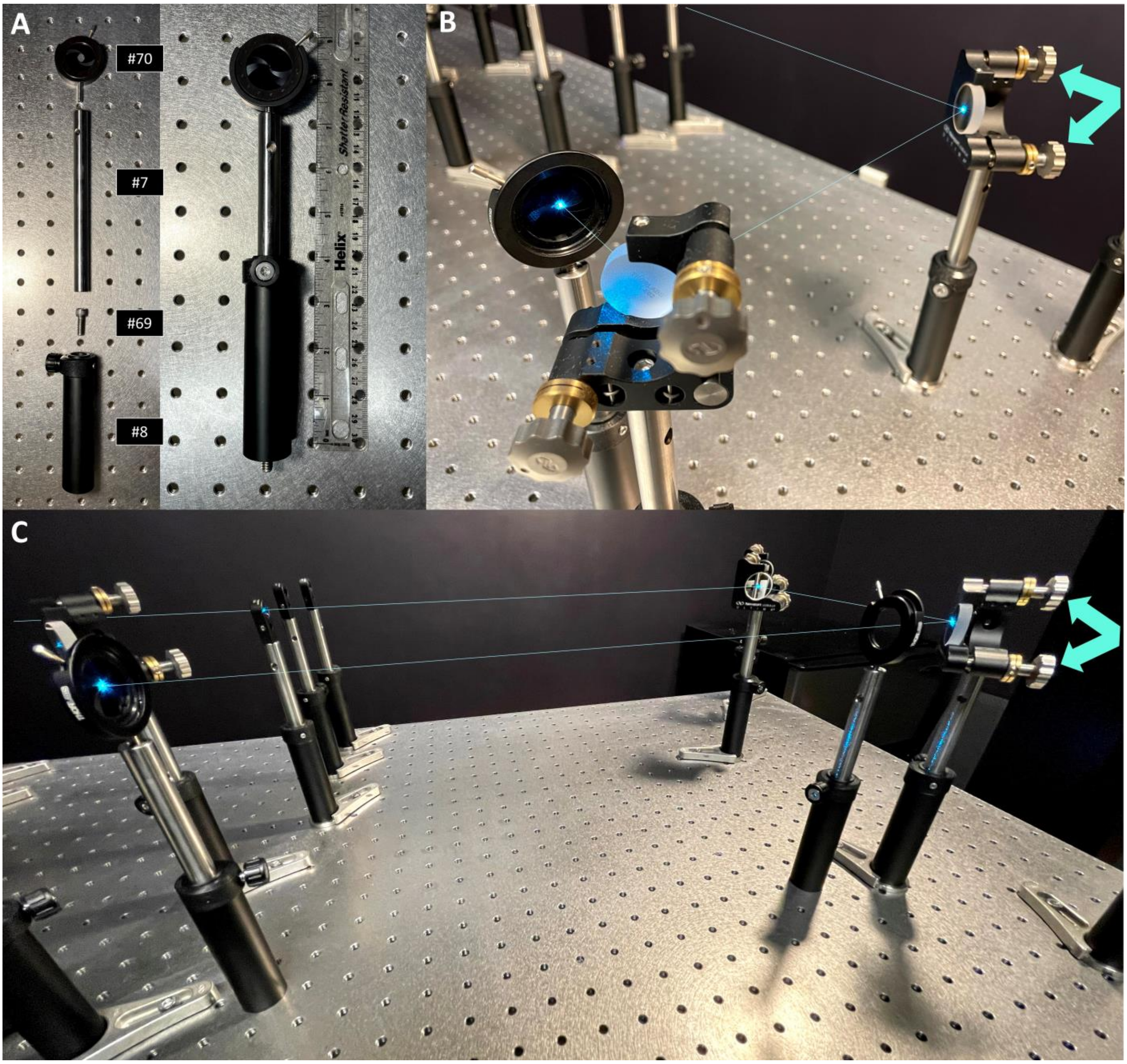

Supplemental Figure 2. Leveling the excitation beams. (A) Leveling beams using a two-mirror turn requires two irises. Each iris is attached to a 6″ post and an adjustable post holder. Before inserting the post into the post holder, a ¼″-20 screw is secured into the bottom of the post holder so the post holder can be mounted directly into the holes of the optical table. To make an 8″ iris assembly, the post is adjusted within the post holder such that the center of the aperture is 8″ off the surface of the table. A slip-on post collar (table 1, #71) can be attached to allow for the post to be rotated within the post holder while maintaining the desired height (not shown). (B) To level a beam using a two-mirror turn, the aperture of the first iris is constricted and the adjustment knobs on the mount of the first mirror are tuned until the beam passes through the very center of the first iris. (C) Then, the aperture of the first iris is opened and the adjustment knobs on the mount of the second mirror are tuned until the beam passes through the very center of the second iris. B and C are repeated iteratively until the beam passes through the very center of both constricted irises.

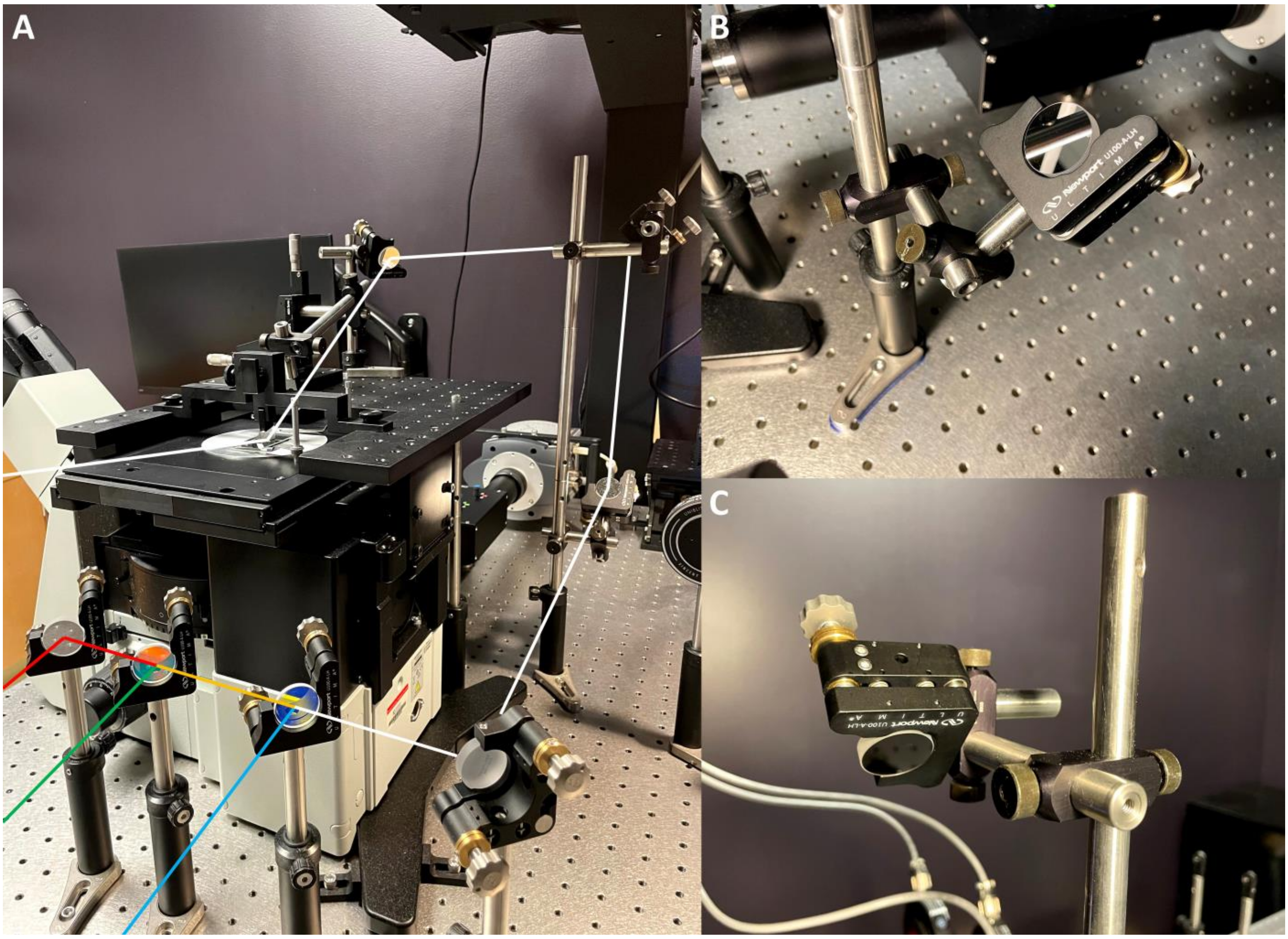

Supplemental Figure 3. Beam path to microscope stage. (A) After the beams are leveled and co-aligned, they are directed to the microscope stage using a series of broadband mirrors. Two broadband mirrors attached to a periscope are used to raise the beam to the level of the microscope stage. The periscope is assembled from three 6″ optical posts, an adjustable post holder, a pedestal base adaptor, and secured to the table near the O26 hole with a slotted clamping fork. (B) The bottom broadband mirror is mounted inside an Ultima clear edge mirror mount, connected to a 2″ post, and attached to the 18″ post with two right angle clamps and a 4″ post. (C) The top broadband mirror is mounted inside an Ultima clear edge mirror mount, connected to a 2″ post, and attached to the 18″ post with two right angle clamps and a 4″ post. The top and bottom mirrors are adjusted iteratively until the beam is directed vertically by the bottom mirror and directed horizontally towards the stage by the top mirror.

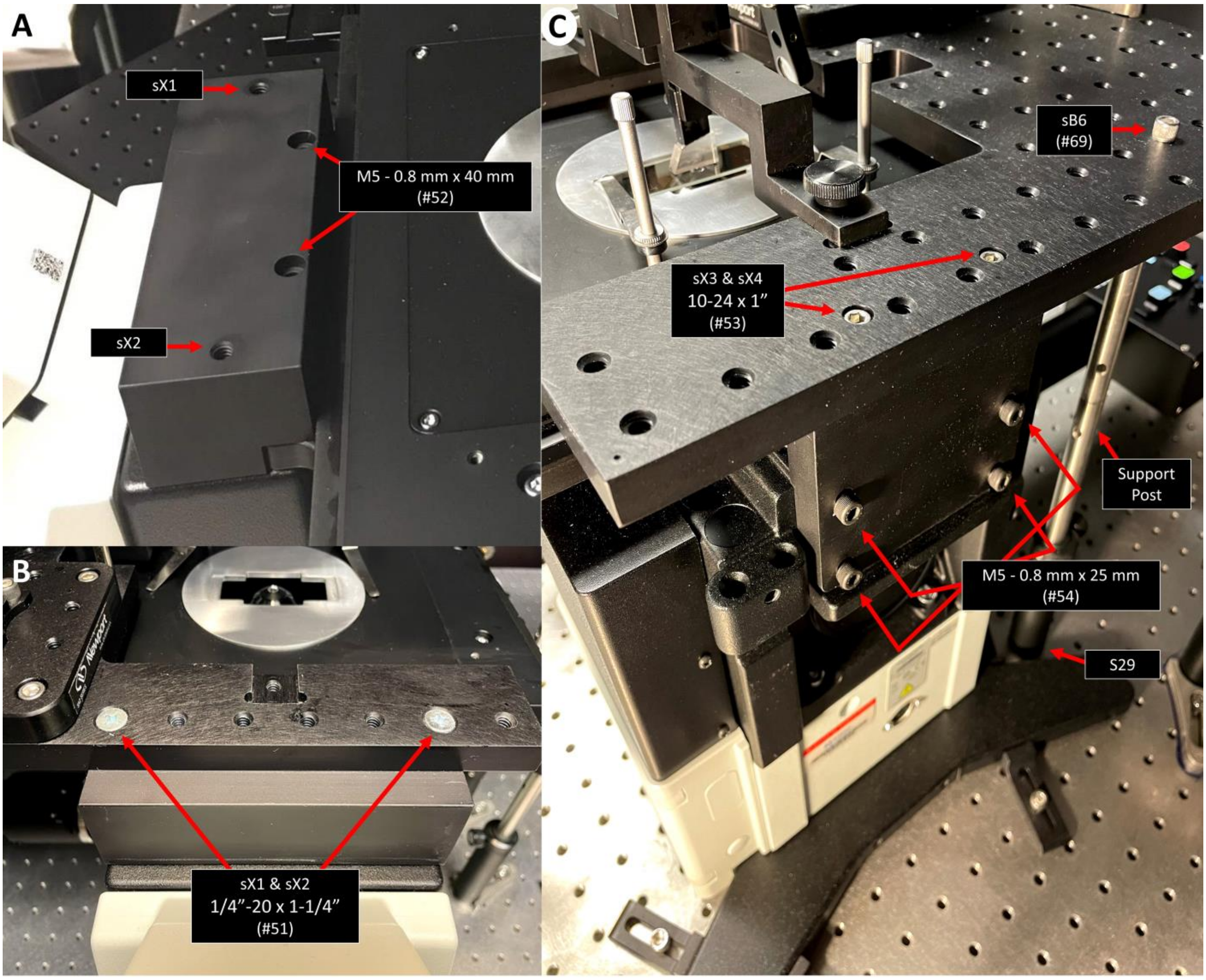

Supplemental Figure 4. Mounting the stage breadboard. (A) The front stage adapter is bolted to the inverted microscope just behind the binocular tube using two 40 mm long M5 screws. (B) The stage breadboard is bolted to the front stage adapter through the sX1 and sX2 holes using two 1-1/4” long ¼”-20 screws. (C) The rear stage adapter is bolted to the back of the inverted microscope using four 25 mm long M5 screws. The stage breadboard is then bolted to the rear stage adapter through the sX3 and sX4 holes using two 1” long 10-24 screws. A support post can be assembled from a 4” post, a 6”post, an adjustable post holder, and a pedestal base adapter and bolted to the stage breadboard through the sB6 hole using a ¼”-20 screw.

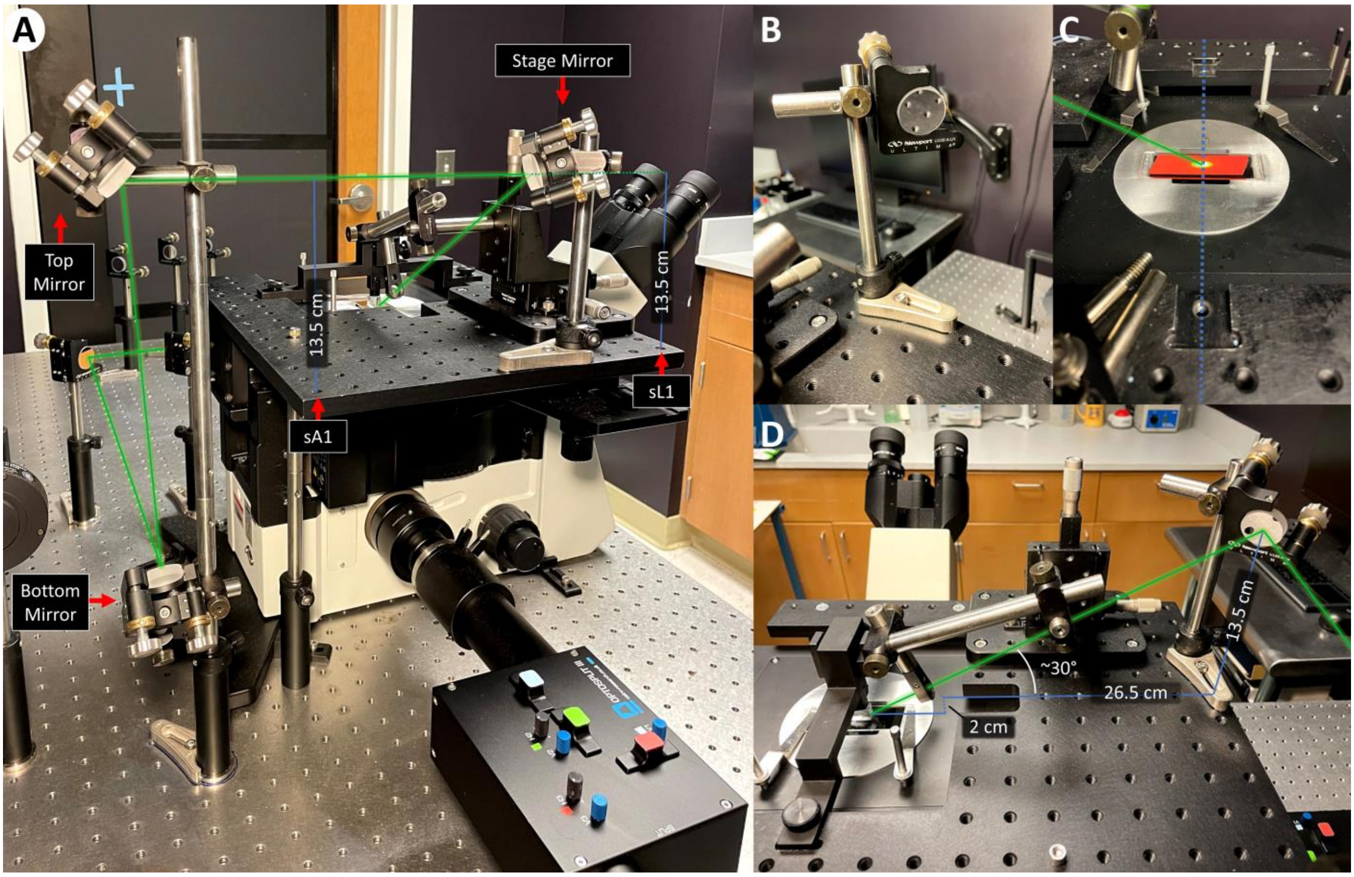

Supplemental Figure 5. Stage mirror and TIRF angle. (A) The top mirror of the periscope is used to direct the beam over the sA1-sF1 holes of the stage breadboard and onto the stage mirror. Before mounting the stage mirror to the stage breadboard, two 13.5 cm tall irises are mounted to the sA1 and sL1 holes of the stage breadboard and the top and bottom mirror are adjusted until the beam passes through the very center of both narrowed apertures. When finished, the irises are removed. (B) The stage mirror is used to direct the beam through the lens and into the prism at the proper angle to induce TIRF. (C) The stage mirror is positioned such that the beam strikes the face of the mirror directly above the sG1 hole, traverses over sG2-sG6, and strikes the microscope stage between the overhead arm mounting holes (sX5 and sX6). (D) The appropriate height at which the beam should strike the stage mirror is dependent on the horizontal distance between the prism and where the beam strikes the stage mirror. In the case of this setup, the horizontal distance between the prism and the reflection point on the stage mirror was measured to be approximately 26.5 cm. Additionally, the microscope stage is approximately 2 cm below the stage breadboard. Thus, the beam should strike the stage mirror 13.5 cm from the surface of the stage breadboard to induce the 30° incident angle at the prism face.

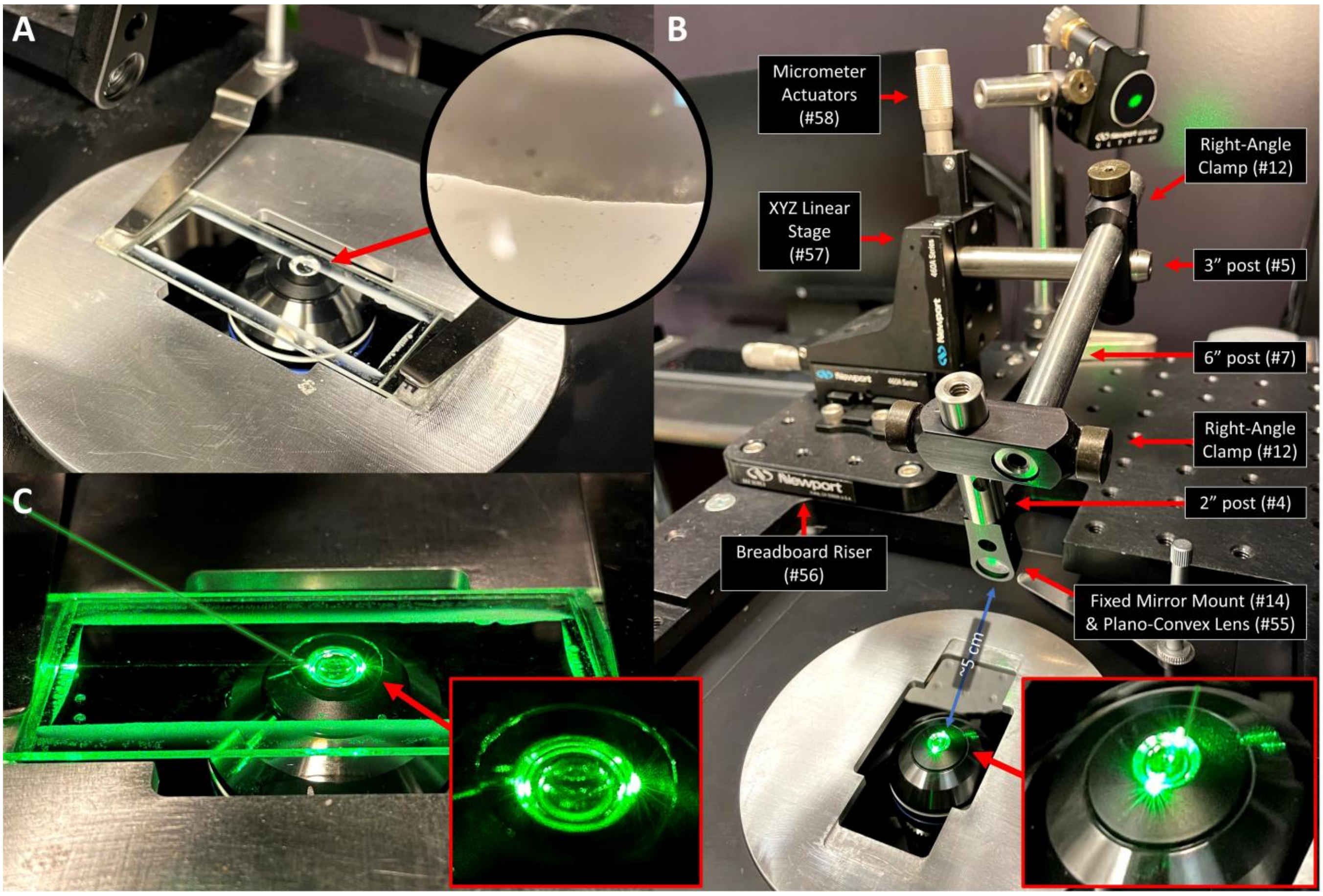

Supplemental Figure 6. Plano-convex lens and XYZ linear stage assembly. (A) The objective is used as a target while setting up the lens assembly and therefore needs to be positioned correctly before assembling the lens and XYZ linear stage. To position the objective, a bead slide is placed on the microscope stage and the inside edge of the chamber is brought into focus. When complete, the objective is at (or very close to) the correct position to observe TIRF in focus within the chamber. (B) The lens assembly consists of a 4″ x 6″ breadboard riser, an XYZ linear stage, three micrometer actuators, two right angle clamps, a 3″ post, a 6″ post, a 2″ post, a fixed mirror mount, and the plano-convex lens. After constructing the lens assembly and mounting it to the stage breadboard, the three micrometers are set to the center of their travel range (~6 mm). Then, the right angle clamps and optical posts are adjusted until the lens is roughly 5 cm from the objective, with the beam passing through the center of the lens and striking the near edge of the objective's front lens. (C) When the lens is positioned properly, the beam will produce symmetric points of light on either side of the objective.

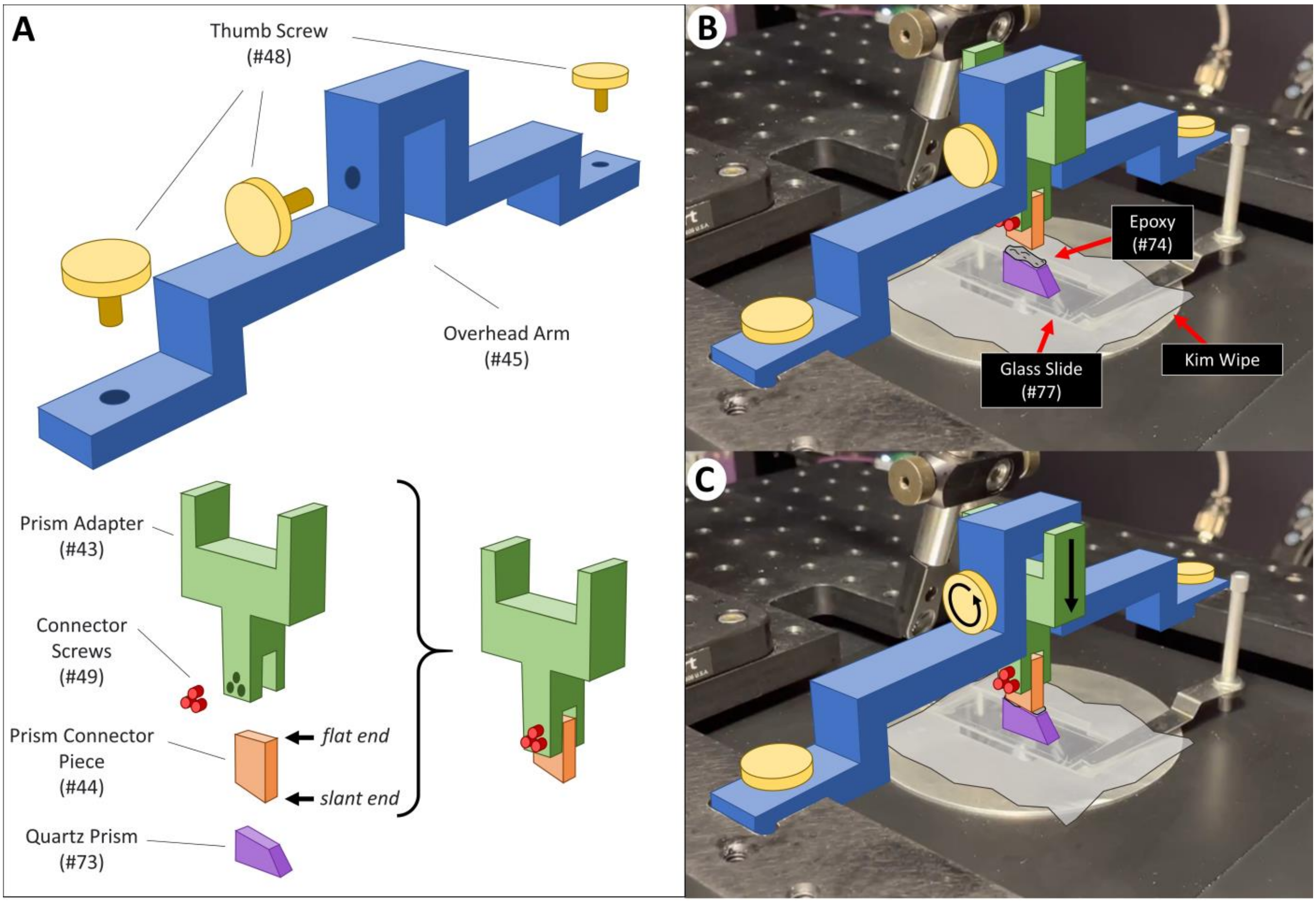

Supplemental Figure 7. Attaching the prism to the prism adapter. (A) The prism assembly consists of a quartz prism, a prism connector piece, three connector screws, a prism adapter, an overhead arm, and three thumb screws. The prism connector piece is inserted into the prism adapter such that the flat end is facing the adapter. The slanted end of the prism connector piece is angled to match the contours of the prism. The prism connector piece is secured to the prism adapter by tightening the three connector screws until they are snug, but not overtightened, which could cause the aluminum adapter to bend. (B) To attach the prism to the prism connector piece, a standard glass slide is secured to the microscope stage using the stage clips and a Kimwipe is laid over the glass slide. The prism adapter is then inserted all the way into the central nook of the overhead arm as shown and the prism adapter is secured in place using the central thumb screw. Then, the overhead arm is attached to the stage breadboard using two thumb screws at sX5 and sX6. A smear of 5-minute epoxy is applied to the top of the prism, and the prism is placed on the Kimwipe directly under the prism connector piece. (C) The central thumb screw is then loosened, and the prism adapter is carefully lowered until the prism connector piece makes contact with the top of the prism. The central thumb screw is then tightened again to secure the prism adapter into place. If needed, a pipette tip can be used to nudge the prism and straighten it with respect to the prism connector piece. The glue is allowed to set overnight. Over a period of use, the prism may become chipped or scratched. When this occurs, the prism can be snapped off of the prism connector and replaced with a new prism.

8

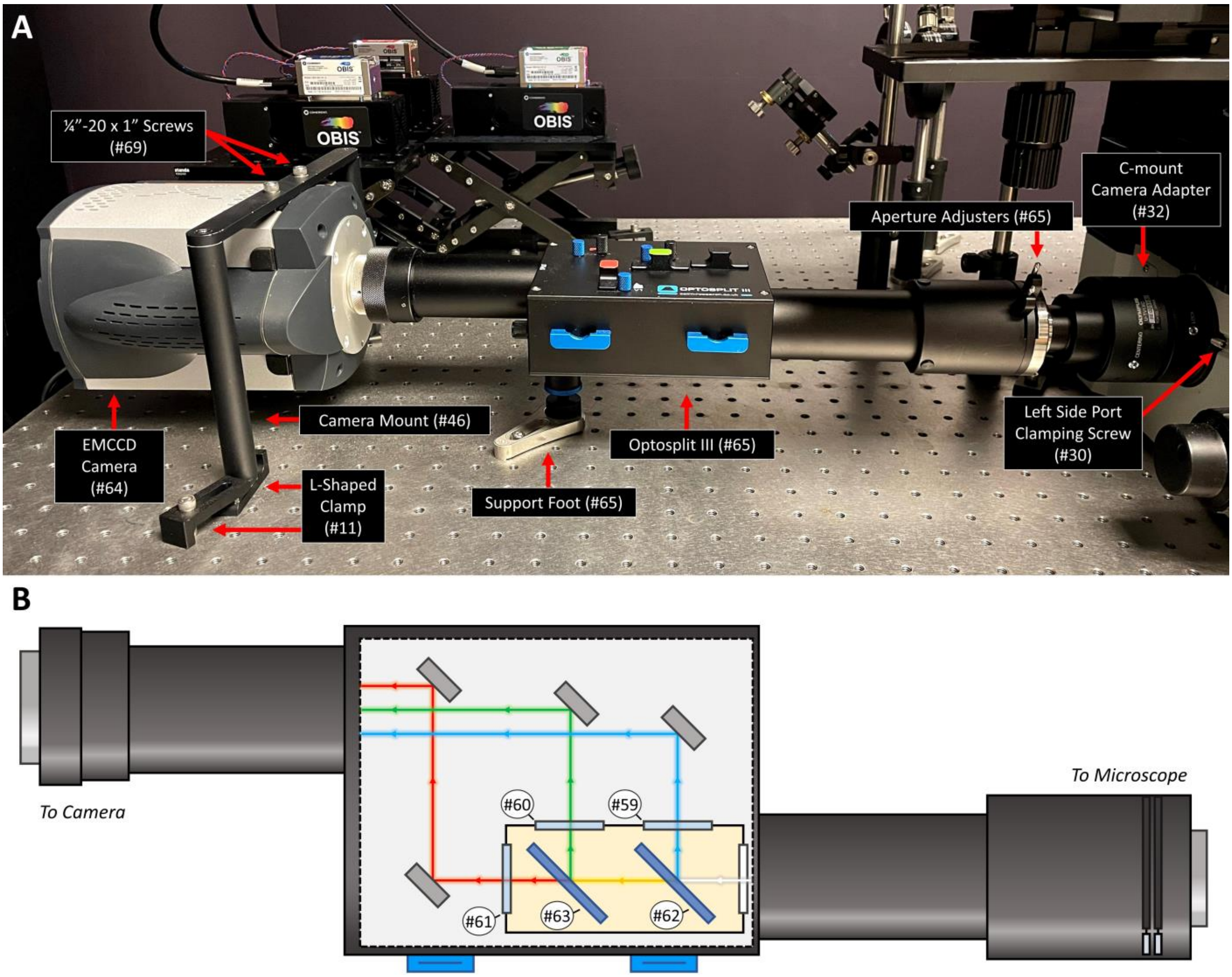

Supplemental Figure 8. Emission path. (A) The emission light path emerges from the left side port of the inverted microscope and enters the Optosplit III, which is connected via the C-mount camera adpater. The EMCCD camera is attached to the custom camera mount which aligns the camera to the exit port of the Optosplit III. (B) The emission filters and dichroic mirrors are mounted within a filter cube (yellow box) that can be removed from the Optosplit III body. The incoming emission light is partitioned first by the 532 nm dichroic (#62), which diverts the AF488 emission through the AF488 emission filter (#59). The remaining light is partitioned by the 635 nm dichroic (#63), which diverts the Cy3 emission through the Cy3 emission filter (#60) and the Cy5 emission through the Cy5 emission filter (#61). Broadband mirrors within the Optosplit III body are used to direct the resulting emission beams to the camera.

9

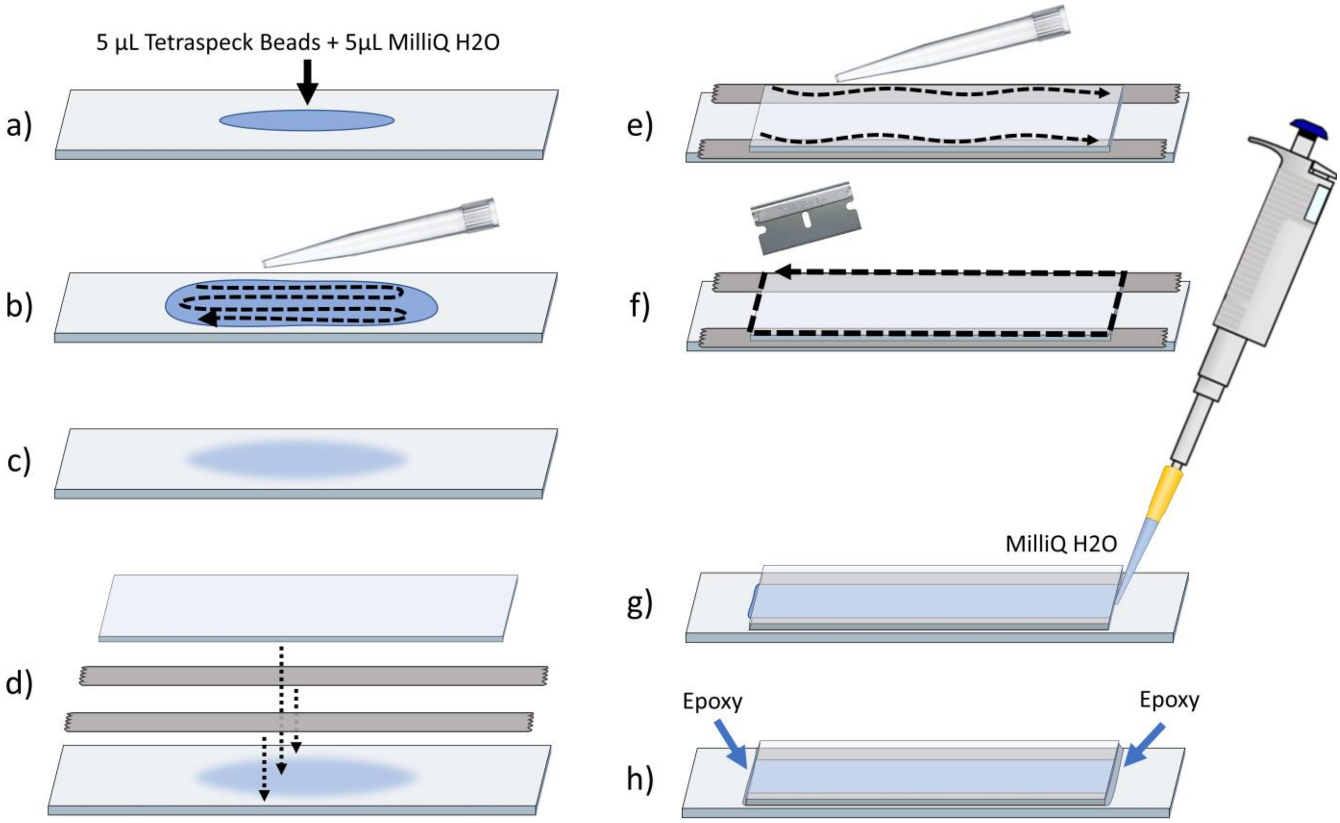

Supplemental Figure 9. Bead slide assembly. (a) 5 µL of concentrated (~2.3 × 10^10 particles/mL) 0.2 µm TetraSpeck™ fluorescent microspheres (table 1, #76) and 5 µL of MilliQ water are pipetted onto the center of a glass slide. (b) The 10 µL volume is spread over the central region of the slide using a pipette tip. (c) The slide is covered and allowed to dry. (d) Two pieces of double stick tape (table 1, #84) are placed across the long edges of the slide on either side of the dried beads and a coverslip (table 1, #85) is placed in the center of the glass slide, on top of the double stick tape. (e) A pipette tip is used to press on the coverslip and seal the bond between the coverslip and the tape. (f) The excess tape surrounding the coverslip is removed with a razor. (g) The space between the coverslip and the slide is filled with MilliQ water. (h) 5-minute epoxy is used to seal the short edges of the enclosure. After 20 minutes, the glue will have set and the slide can be used. A bead slide can last for over a month, but the image quality will eventually depreciate and the slide will need to be replaced.

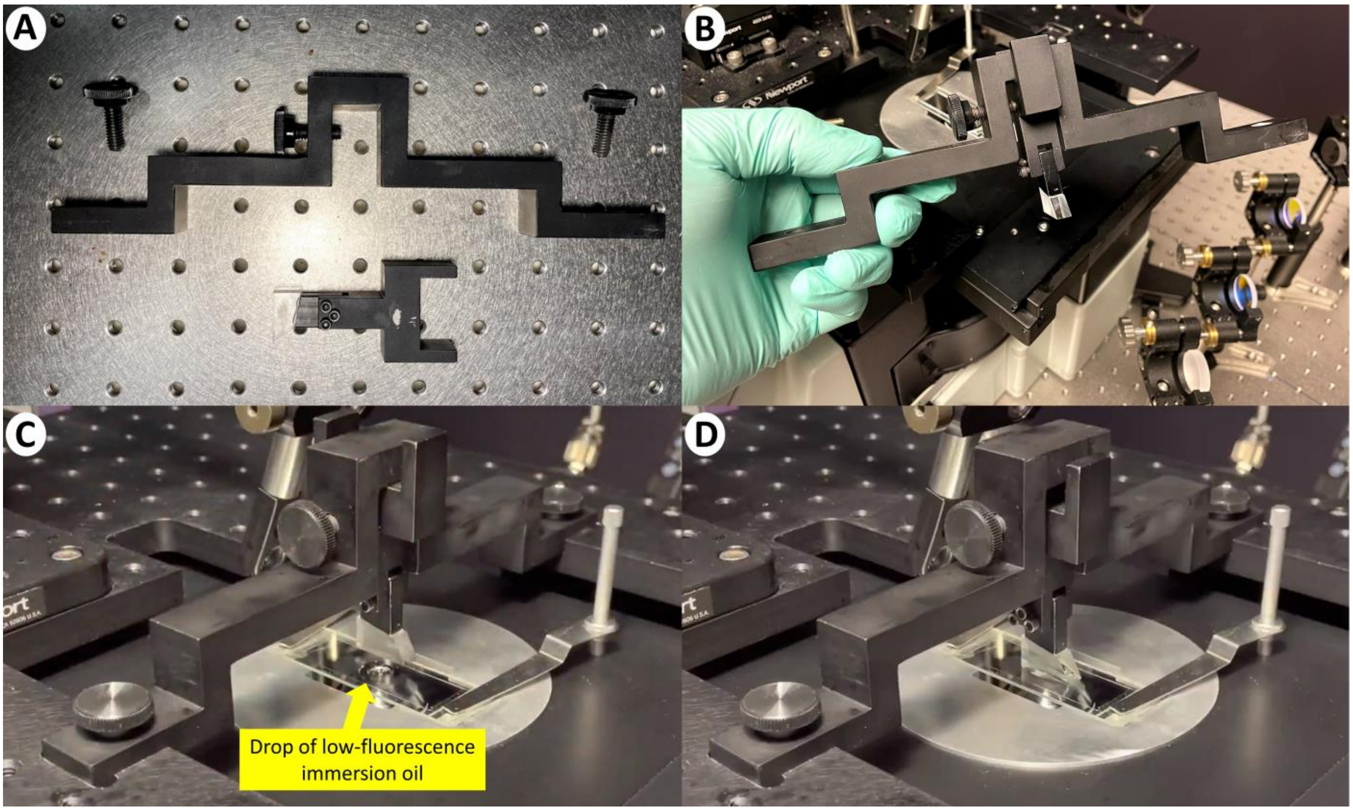

Supplemental Figure 10. Mounting the prism adapter and overhead arm. (A) The prism is held in place above the slide using the prism adapter and overhead arm. (B) To mount the prism assembly onto the microscope during operation, the prism adapter is first inserted all the way up into the central nook of the overhead arm and secured into place by tightening the central thumb screw. (C) After securing the slide to the microscope stage with the stage clips and placing a drop of low autofluorescence immersion oil on the slide, the overhead arm is inserted into the slots of the stage breadboard and secured in place by tightening the thumb screws at sX5 and sX6. (D) After the overhead arm is secured to the stage breadboard, the top of the prism adapter is held with one hand to prevent it from dropping, while the other hand loosens the central thumb screw. With the prism adapter free, the prism is gently lowered onto the drop of oil and secured in place by retightening the central thumb screw. To remove the slide, the prism assembly must first be removed by performing the above steps in reverse.

11

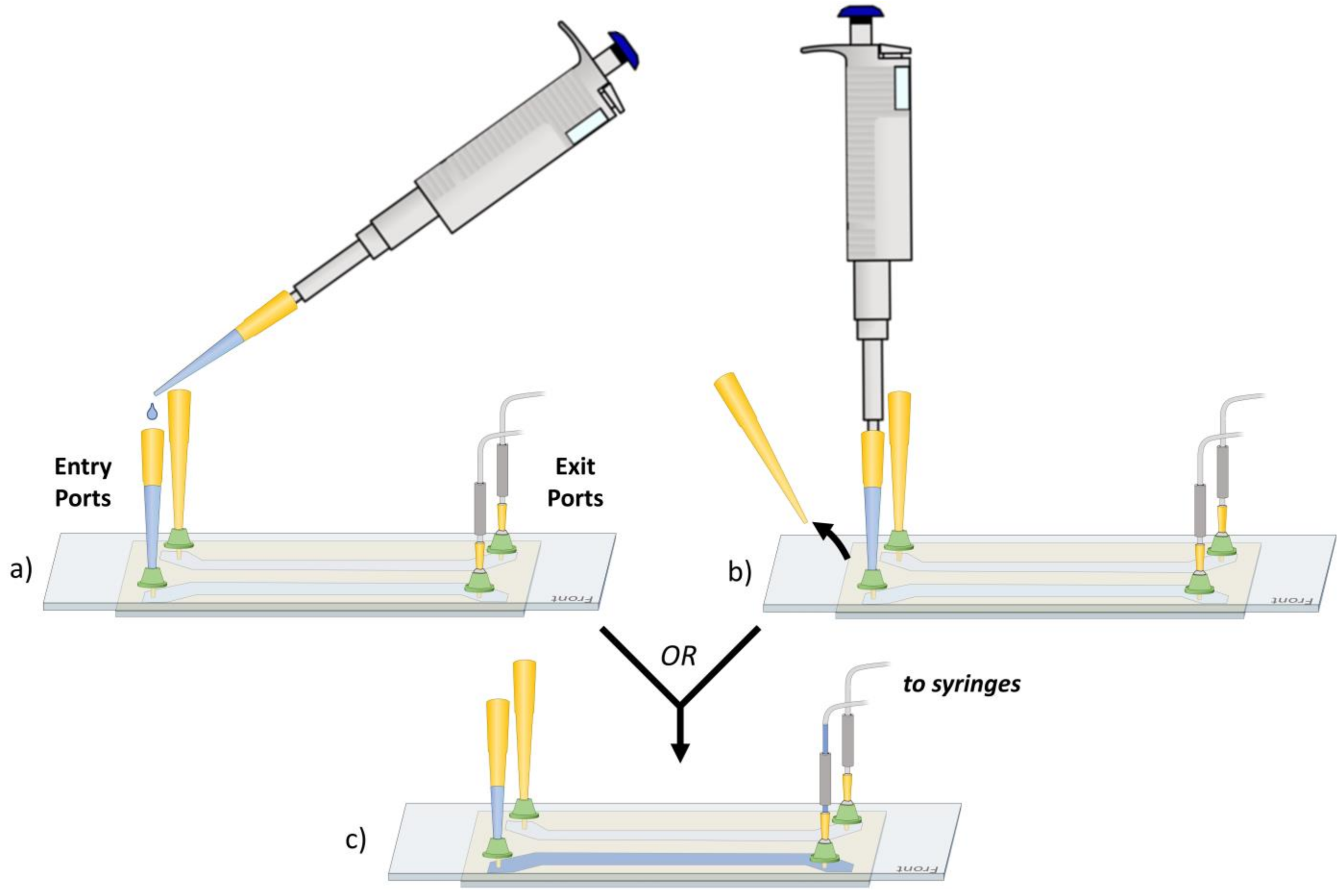

Supplemental Figure 11. Using a sample chamber. (a) To allow for easy and secure insertion of pipette tips into the holes of the sample chamber, we have designed 3D printable ports which can be glued to the surface of the slide. The entry ports located on one side of the chamber are used to support 200 μL pipette tips, which serve as replaceable reservoirs for solutions. The reservoirs can be filled by pipetting directly into the top of the pipette tip. (b) Alternatively, the reservoir can be replaced by removing the old tip from the entry port, pulling fresh solution into a new tip with a pipettor, inserting the tip into the empty entry port, and ejecting the tip from the pipettor. (c) The solution in the reservoir can then be pulled through the sample chamber by pulling negative pressure on the syringe attached to the exit port on the opposite side. Care should be taken to avoid flowing air through the sample chamber, which can cause issues with the immobilized substrates and/or fluorescent molecules within the chamber.

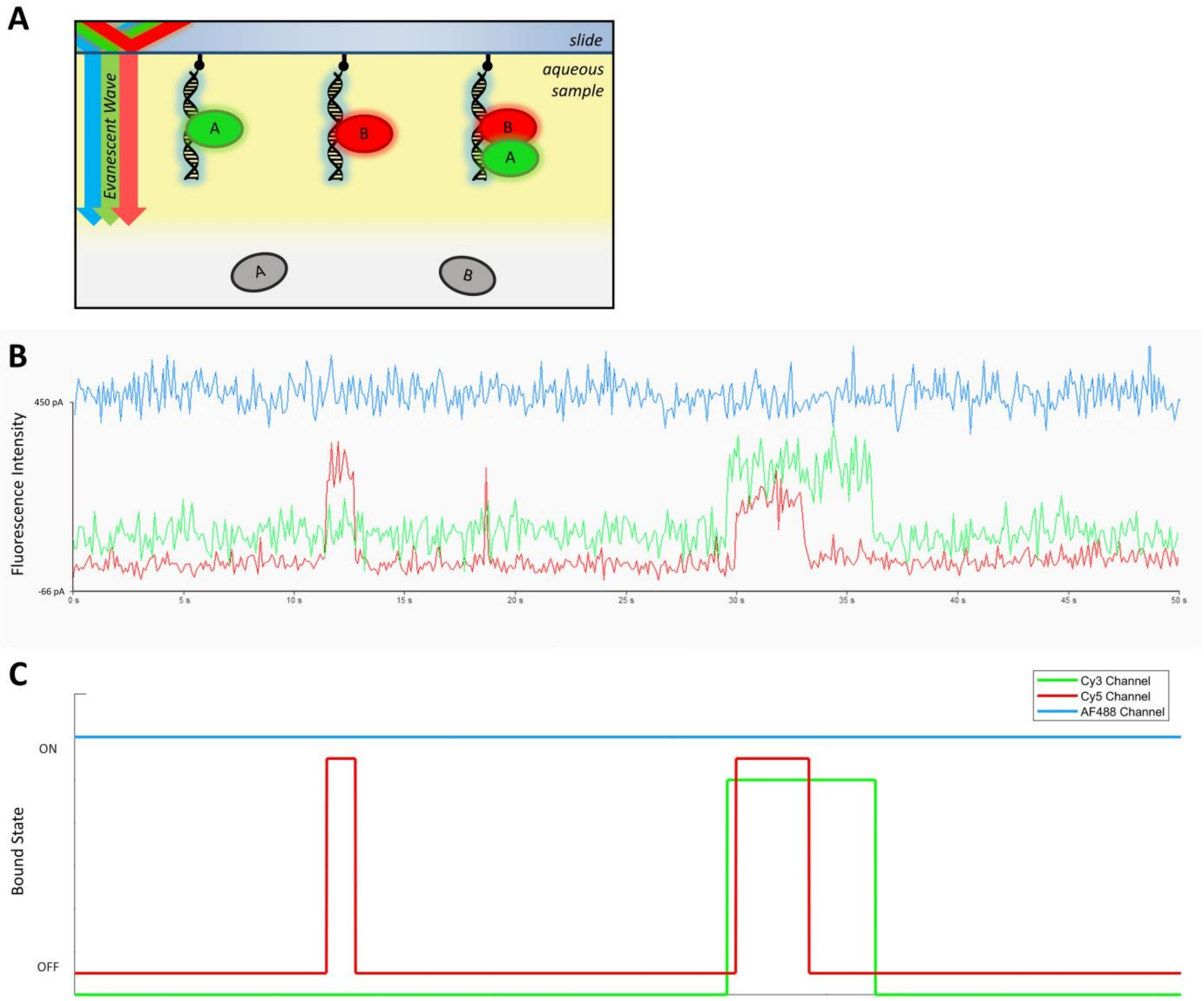

Supplemental Figure 12. Representative Data (A) An example of a three-color single-molecule TIRF colocalization experiment that can be carried out using a three-color prismTIRF microscope is shown. In this example, AF488-labeled biotin-DNA (blue glow) is immobilized to the surface of the slide and Cy3 ("A") and Cy5 ("B") labeled DNA binding proteins are flowed into the sample chamber. (B) A representative trajectory of a single AF488-labeled DNA molecule (blue) and interacting Cy3 (green) and Cy5 (red) labeled DNA binding proteins. (C) Trajectories are idealized into descrete states (e.g. ON/OFF) and imported into KERA, which can be used for statistical analysis of large collections of events. Short spikes in fluorescence lasting less than 300 milliseconds result from fluorescent molecules flowing past the imaged region and are disreguarded.
